# Development of evolutionarily conserved viral integration sites as safe harbors for human gene therapy

**DOI:** 10.1016/j.isci.2025.113910

**Published:** 2025-11-01

**Authors:** Marco A. Quezada-Ramírez, Matthew A. Campbell, Krishna M. Parsi, Robert J. Gifford, Robert M. Kotin

**Affiliations:** 1 Department of Genetic and Cellular Medicine, University of Massachusetts Chan Medical School, Worcester, MA, USA; 2 University of Alaska Museum of the North, University of Alaska Fairbanks, Fairbanks, AK, USA; 3 Department of Animal Science, University of California, Davis, Davis, CA, USA; 4 MRC-University of Glasgow, Centre for Virus Research, Bearsden, Glasgow, UK; 5 Center for Biomedical Innovation, Massachusetts Institute of Technology, Cambridge, MA, USA

**Keywords:** genomics, genetic engineering, stem cells research

## Abstract

Gene transfer into CD34^+^ hematopoietic stem and progenitor cells (HSPCs) involving integrating viral vectors has unpredictable outcomes including potential severe events like leukemogenesis, resulting from insertional mutagenesis. Therefore, identifying and characterizing genome safe harbor (GSH) sites where exogenous genetic material can be safely integrated is critically important for therapeutic gene addition. Here, we present an approach to identify GSH candidates based on a proven system of stable transgene insertion: the evolutionarily conserved integration of parvoviral DNA into the germlines of host species. By analyzing the preservation of endogenous parvoviral elements (EPVs) from phylogenetically diverse vertebrate genomes, 102 EPV loci were mapped to the human genome, and 17 underwent experimental evaluation as GSHs in CD34^+^ HSPCs. This resulted in at least six loci tolerant to transgene insertion, low transcriptome disturbances, and three loci showing myeloid immune branch-specific regulation. Thus, our approach produced a catalog of candidate GSHs suitable for therapeutic transgenesis in human cells.

## Highlights

• Genome safe harbor sites must guarantee safe and stable transgene insertion
• Endogenous parvoviral elements (EPVs) resulted from ancient integrative infections
• Preserved EPVs into the human genome indicate places tolerant to gene addition
• Tissue specific-GSH regulation increases the safety of therapeutic gene addition

## Graphical abstract

**Figure:**
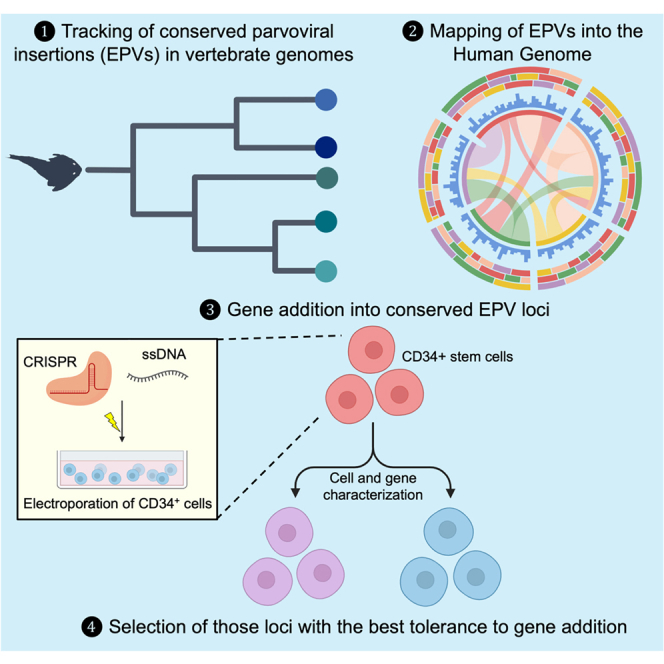

## Introduction

Genome safe harbors (GSHs) are defined as sites where therapeutic transgenes can be introduced without disrupting normal cellular functions while maintaining stable and predictable transgene expression upon cellular division. In contrast, gene therapy applications that utilize integrating viral vectors, e.g., lentivirus or gammaretrovirus, tend to integrate indiscriminately into transcriptionally active, and accessible euchromatin regions, with potential consequences resulting in genotoxicity, gene silencing, unpredictable expression, and actual risk of malignancy, e.g., leukemia.^1^^,^^2^^,^^3^ Thus, identifying GSHs that provide reliable locations for therapeutic gene addition becomes a high priority for gene therapies utilizing lentiviral transduction to address blood disorders like multiple myeloma,^4^ β-hemoglobinopathies,^5^^,^^6^ coagulopathies,^7^ immunodeficiencies,^8^^,^^9^ or anemias.^10^

Currently, efforts are ongoing to find human GSH sites beyond the widely applied *AAVS1*,^11^^,^^12^ *CCR5*,^13^^,^^14^ and *hROSA26*^15^^,^^16^ loci. Proposed criteria for identifying GSHs include the following: >50 kb distance from the 5′ end of any gene, or >300 kb from cancer-related genes (or microRNAs), and outside of transcription units (or ultra-conserved regions).^17^^,^^18^^,^^19^^,^^20^ However, the three established GSH loci violate these criteria and the number of characterized sites that follow these rules have been limited and mostly evaluated in established cell lines with few examples in primary human cells.^21^^,^^22^ Alternatively, the insertion into endogenous mobile elements,^23^ rDNA sequences,^24^ or based on high-order chromatin architecture analysis^25^^,^^26^ has not been particularly effective. Thus, the *AAVS1,* remains a popular and widely used site for directed transgenesis^27^ given that the knock-in neither affects cell viability nor interferes with differentiation.^28^^,^^29^ However, some reports observed transgene silencing occuring in a cell lineage,^30^ or promoter-specific manner.^31^ The *AAVS1* locus was identified as an AAV provirus integration site in a region spanning the first exon and the first intron of *PPP1R12C* on human chromosome 19 (within a motif containing an AAV minimum, Rep-dependent, origin of replication),^11^^,^^12^^,^^32^ although most of the commercially available editing reagents target into the PPP1R12C first intron.^33^ The use of *AAVS1* as a GSH was well established,^34^^,^^35^^,^^36^ and given that the *AAVS1* arose as a parvovirus integration site we hypothesized that other parvoviral integration loci may also be predictive of GSH sites.

Parvoviruses are among the small number of viruses known to have contributed genes to the vertebrate germline via viral integration and horizontal gene transfer.^37^ Consequently, endogenous parvoviral elements (EPVs) are the result of ancient infections across phylogenetically diverse host species that were preserved during multiple speciation events through geologic timescale.^38^ An extensive phylogenetic analysis of EPVs in vertebrate genomes disclose clear homology to members of extant parvovirus genera, including *Amdoparvovirus*,^39^ *Protoparvovirus*,^40^^,^^41^ and *Dependoparvovirus*.^38^^,^^42^ The viral DNA endogenization is a a large-step mutation, or saltation, event resulting in the acquisition of viral “alleles” that may have disrupted gene expression, introduced *cis* acting motifs (such as transcription factor binding sites, promoters, and splicing signals), or resulted in “exaptation” of viral genes that potentially benefited the host organisms.^43^^,^^44^ Thus, identifying human orthologs of the EPV loci in non-human vertebrate genomes might indicate genomic loci tolerant to the gene addition.

In this study, we identified 102 human orthologs of EPVs from a variety of endothermic host species as potential human GSHs.^45^ Orthologous loci were broadly categorized as intergenic or intronic according to the relative location in the host species and in the human genome. Seventeen of these loci (8 intergenic and 9 intronic) were experimentally evaluated by inserting an *eGFP* expressing cassette into primary human CD34^+^ hematopoietic stem and progenitor cells (HSPCs), a clinically relevant *in vitro* model. The edited cells displayed no alterations in self-renewal or multipotency (i.e., stemness) in cell culture, demonstrating tolerance to the gene addition. Moreover, the transgene expression was maintained across differentiation stages, indicating stable integration and expression. RNAseq analysis revealed that transgene insertion, and expression, resulted in minimal transcriptome disturbances from six loci. Remarkably, some GSH loci displayed myeloid immune restrictive regulation. To our knowledge, this is the first study that provides a catalog of human GSH sites with potential applications for CD34^+^ HSPC-based gene therapies. Furthermore, it is likely that lineage restrictive expression is not exclusive to HSPCs and can be extended to other tissues and additional GSHs likely emerging from the remaining mapped loci.

## Results

### EPVs are evolutionary preserved in the human genome

**Figure 1:**
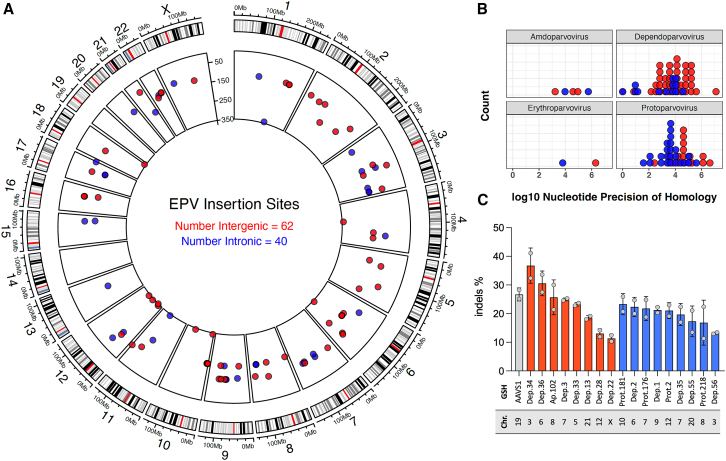
Evolutionary preserved EPV loci in the human genome represent 102 potential GSH sites (A) Circle plot of EPV orthologues mapped into the human genome. The outer layer represents human chromosomes, 1 to 22, and X chromosome lengths are indicated by the tick marks, e.g., 0–100 Mb. Black and red radial marks represent chromosomal banding patterns and centromeres, respectively. The inner layer depicts intergenic and intronic locations of EPVs (i.e., potential GSHs) as red and blue circles, respectively, with the estimated divergence time of the host species taxa from humans scaled from 0 to 350 million years ago. (B) Comparative genomic analysis of the precision of homology between EPV host species and human genome. EPVs are derived from four parvovirus genera: *Amdoparvovirus*, *Dependoparvovirus*, *Erythroparvovirus*, and *Protoparvovirus*. The distribution displays abundance of *Dependoparvovirus-* and *Protoparvovirus-*related loci. Intergenic and intronic EPVs correspond to the red and blue circles, respectively, showed in (A). (C) Propensity to genome editing of 17 human EPV orthologs evaluated in HEK293T cells after the expression of *spCas9* and the corresponding gRNA. Intergenic or intronic categories are depicted as red and blue bars, respectively. Targeting of the EPV-derived *AAVS1* locus (gray) is shown for comparison. The nomenclature for the assessed GSH sites (abbreviated EPV name) as well as the chromosome hosts is indicated on the *x* axis. Indel frequencies were determined through TIDE decomposition analysis. Mean ± SD, *n* = 2 independent experiments.

The local genomic landscape surrounding EPV insertions may be broadly categorized as intergenic or intronic according to the equivalent orthologous position in the human genome. A total of 102 mammalian EPV loci (62 intergenic and 40 intronic) were mapped in the human genome by comparative genomics, regional genome alignments, and similarity of DNA flanking EPVs using BLAT or BLASTN analysis (Figure 1A). Relative positions between host species and human genome were established by interspecific collinearity and were refined to more precise coordinates based on sequence homology (Table S1).

The putative GSH loci identified via this approach were found distributed across the human genome without obvious sequence or higher order common features (Figure 1A; Table S1). The majority of the EPVs were derived from members of the *Dependoparvovirus,* generally considered to be apathogenic, and *Protoparvovirus* genera, often disease causing viruses (Figure 1B). An EPV locus nomenclature referencing the parvoviral genus from which each EPV is derived was established for this report and can be consulted together with the coordinates of their relative position in the human genome, as well as the identity of the flanking genes (or host genes for intronic loci) in Tables 1 and S1.

**Table 1:** Human GSH sites experimentally assessed in primary CD34^+^ HSPCs

EPV nomenclature^a^	Abbrev. name	Assigned category	Flanking genes or host gene	Human coordinates of EPV^b^	ATAC peak to^c^
*Dependo.34-megadermatvespertilion*	*Dep.34*	intergenic	*RPL18AP8/RCC2P5*	Chr3: 96,719,524-96,721,025	6.0 kb
*Dependo.36-phyllostomidae*	*Dep.36*	intergenic	*TMEM200A/SMLR1*	Chr6:130,764,851-130,765,531	10.0 kb
*Ap.102-procavia*	*Ap.102*	intergenic	LINC00824*/CCDC26*	Chr8:128,435,359-128437048	29.69 kb
*Dependo.3-lagomorpha*	*Dep.3*	intergenic	*NUP42/GPNMB*	Chr7:23,218,501-23,222,555	0.55 kb
*Dependo.33-pteropodidae*	*Dep.33*	intergenic	*PARP8/ISL1*	Chr5:50,957,501-50,959,300	0.47 kb
*Dependo.13-cercopithecidae*	*Dep.13*	intergenic	*HUNK/Mis18A*	Chr21:32,097,568-32,098,575	90.0 kb
*Dependo.28-daubentonia*	*Dep.28*	intergenic	*LRRK2/CNTN1*	Chr12:40,658,392-40,660,499	31.64 kb
*Dependo.22-laurasiatheria*	*Dep.22*	intergenic	*DIAPH2/PCDH19*	ChrX:98,175,245-98,175,812	16.0 kb
*AAVS1*	*AAVS1*	intronic	*PPP1R12C*	Chr.19:55,112,144-55,117,873	0.26 kb
*Proto.181-PhaCin*	*Prot.181*	intronic	*MANCR*	Chr10:4,652,078-4,656,006	32.67 kb
*Dependo.2-vespertilionidae*	*Dep.2*	intronic	*KIF6*	Chr6:39,422,982-39,425,107	15 kb
*Proto.176-NanGal*	*Prot.176*	intronic	*POT1-AS1*	Chr7:125,056,326-125,057,364	5.14 kb
*Dependo.1-whippomorpha*	*Dep.1*	intronic	*PAX5*	Chr9: 36,852,789-36,855,146	0.87 kb
*Proto.2-MusSpr*	*Prot.2*	intronic	*PDZRN4*	Chr12:41,262,344-41,264,604	1.28 kb
*Dependo.35-vespertilionidae*	*Dep.35*	intronic	*ANKRD7*	Chr7:118,262,500-118,263,554	24.6 kb
*Dependo.55-rodent*	*Dep.55*	intronic	*BCAS4*	Chr20:50,799,100-50,801,360	4.0 kb
*Proto.218-VomUrs*	*Prot.218*	intronic	*ENPP2*	Chr8:119,591,710-119,593,739	3.74 kb
*Dependo.56-cavia*	*Dep.56*	intronic	*GUCA1C*	Chr3:108,912,857-108,913,858	1.14 kb

a The nomenclature to identify human orthologs of EPVs refers the parvoviral genus of origin. An abbreviated name is indicated in the second column. For intergenic GSH sites, the flanking genes are indicated. When the candidate GSH falls in an intron, the host gene is indicated.

b Human coordinates correspond to the sequences retrieved from Ensembl to engineer the gRNAs.

c The nearest ATAC peak (open chromatin spot) per candidate GSH site was determined with Integrative Genomics Viewer (IGV) using the results informed by Corces et al.^46^

### GSH candidates show a propensity to genome editing similar to the reference *AAVS1* locus

Seventeen loci (8 intergenic and 9 intronic) were selected for a first round of screening in HEK293T cells (Table 1; Figure 1C). The loci proximity to open chromatin regions was evaluated with available ATAC-seq data for human CD34^+^ HSPCs.^46^ Then, single-guide RNAs (hereafter gRNAs) were designed using online predictive tools with three to five highly scored gRNAs per locus incorporated into pX330 plasmids to co-express together with *SpCas9*. HEK293T cells were independently transfected to select the most effective gRNA per locus compared to a widely cited gRNA targeting the reference GSH, *AAVS1*^47^ (Figure 1C). Relative to *AAVS1*, all the GSH candidates showed reasonable propensity to genome editing (Figure 1C); therefore, these loci were then evaluated by editing primary CD34^+^ HSPCs obtained from human healthy donors.

### GSH sites are accessible to transgene insertion in human CD34^+^ HSPCs

**Figure 2:**
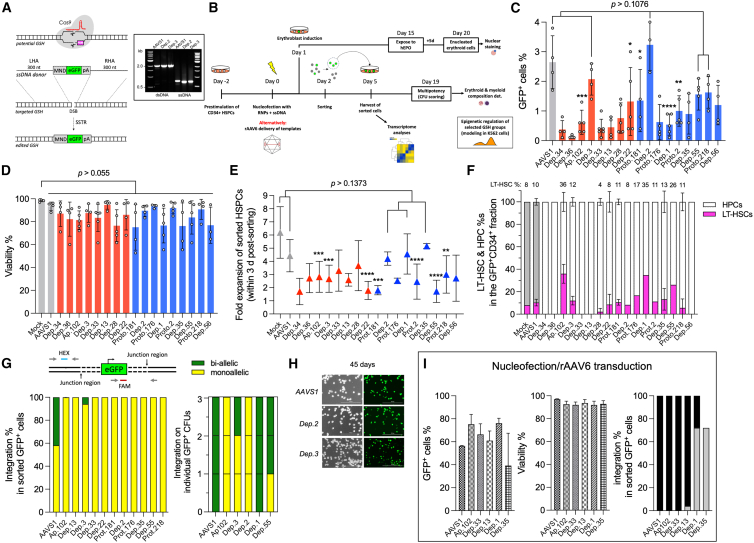
Candidate GSH sites are accessible to transgene insertion in primary human CD34^+^ HSPCs resulting in stable gene addition (A) Genome targeting with *eGFP*-expressing cassettes into GSH candidates using locus-specific CRISPR-Cas9-ssDNA editing sets. Single-stranded DNA donor templates obtained from duplex substrates (inset) were flanked with 300 nt homology arms to mediate HR through the single-stranded template repair pathway (SSTR). Other templates are available in Figure S1A of the supplemental information. (B) Experiments with bone marrow derived CD34^+^ HSPCs. Prestimulated cells were nucleofected in presence of the CRISPR-Cas9-ssDNA editing set, and cultured for two days prior to sorting the GFP^+^ cells. The GFP^+^ cells were cultured for an additional three days before downstream processing. Alternatively, rAAV6 vectors were used to deliver templates to target *Ap.102*, *Dep.33*, *Dep.13*, *Dep.1*, and *Dep.35* loci. In parallel, 1 day post-nucleofected cells were cultured in erythroblast expansion conditions during 15 days before the maturation with human EPO. (C) Percentage of GFP^+^ cells at two days post-nucleofection. Intergenic and intronic loci are depicted as red and blue bars, respectively. Open circles represent individual human donors. *AAVS1* GSH (gray) served as the reference control. Mean ± SD, ANOVA followed by Dunnett test ∗*p* < 0.05, ∗∗*p* < 0.01, ∗∗∗*p* < 0.001, and ∗∗∗∗*p* < 0.0001. *n* ≥ 4 independent experiments. (D) Cell viability determined by flow analysis with propidium iodide treatment. Controls correspond to unedited cells (mock). Open circles represent independent human donors. Mean ± SD, ANOVA followed by Dunnett test, *n* ≥ 3 independent experiments. (E) Proliferation of GFP^+^ cells within the three days post-sorting (fold expansion). ANOVA followed by Dunnett test. ∗∗*p* < 0.01, ∗∗∗*p* < 0.001, and ∗∗∗∗*p* < 0.0001. *n* ≥ 3 independent experiments. (F) Targeting of LT-HSC (CD34^+^/CD90^+^, magenta) and progenitor populations (CD34^+^/CD38^+^, CD34^+^/CD38^−^) within the GFP^+^/CD34^+^ modified cell fraction. *n* = 2–3 independent experiments with different human donors. Percentages of LT-HSCs are showed for the most promising GSH candidates. (G) Targeted integration in the pool of GFP^+^ cells sorted after 14 days of liquid culture (left). Locus-specific primer/probe sets are depicted at the top. Determinations on individual differentiated colonies are shown for *Dep.1*, *Dep.2*, *Dep.3*, *Dep.55*, and *Ap.102* targeted cells (left). The integration percentages were calculated as the FAM/HEX ratio of fluorescent probe signals from *n* = 2 independent pool of cells, or 3 individual colonies, respectively. (H) Persistent transgene expression after 45 days of liquid culture. Phase contrast and fluorescent images are shown for *AAVS1* (reference), *Dep.2* (intronic), and *Dep.3* (intergenic) GSHs. Scale bars, 200 μm. (I) Electroporation with gRNA:Cas9 complexes followed by rAAV6 transduction of templates for *AAVS1* (reference), *Ap.102*, *Dep.33*, *Dep.13*, *Dep.1*, or *Dep.35* loci. Increased levels of GFP^+^ HSPCs with no effects on cell viability are observed in addition to high extent of bi-allelic insertion (black bars) for most of the loci. Mean ± SD, *n* = 2 independent experiments.

Gene targeting into HSPCs has been reported using plasmids or linear double-stranded DNA (dsDNA) carrying homology arms to mediate the homologous recombination (HR)^21^^,^^48^ or flanked with gRNA recognition sites for an homology-independent targeted integration (HITI).^49^ However, such templates elicit robust innate immune responses increasing the cytotoxicity and are transcriptionally active without integration. On the other hand, the use of rAAV6 vectors on HSPCs enhance the editing outcomes,^9^ but the viral vector DNA tends to remain episomal after transduction with positive and negative strands potentially annealing into transcriptionally active dsDNA, leading to false positive results. Thus, we opted for single-stranded DNA (ssDNA) templates as these reportedly correct point mutations associated to blood disorders (Figure 2A).^50^^,^^51^ Comparing dsDNA and ssDNA templates, the ssDNA templates displayed reduced eGFP background (Figures S1A–S1D) and may also reduce the off-target integration through stochastic, cell-mediated recombination, and false positives resulting from unintegrated templates (Figures S1E and S1F). Consequently, we engineered ssDNA templates for each locus composed of the *eGFP* coding sequence regulated by the MND synthetic promoter and the rabbit beta-globin polyadenylation signal (Figure 2A). Every cassette was flanked by site specific-300 nt homology arms yielding 2 kb templates.

Synthetic gRNAs complexed with SpCas9 were then codelivered with the corresponding ssDNA template into 2.5 × 10^5^ primary human CD34^+^ HSPCs using nucleofection (i.e., electroporation). To minimize transcriptional interferences, intronic GSH-directed templates were engineered to insert the transgene in opposite orientation relative to the transcriptional unit hosting the candidate GSH site. Two days after nucleofection, the GFP^+^ cells were sorted and cultured for an additional 3 days (Figure 2B). Unless noted differently, the experiments with undifferentiated CD34^+^ HSPCs spanned seven days to avoid impairing the stemness features that occur following prolonged cell culture.^52^ The GFP^+^ percentages ranged from 0.12% ± 0.05 GFP^+^ cells (mean ± SD, *p* < 0.0001) for the intergenic locus *Dep.36*-targeted cells, up to 3.23% ± 0.86 GFP^+^ cells (*p* = 0.8938) for the intronic locus *Dep.2* (host gene *KIF6*), with several intermediate groups (Figure 2C). The reference GSH, *AAVS1*, reached 2.65% ± 0.88 GFP^+^ cells. Not surprisingly, the GFP^+^ percentages in CD34^+^ cells differed substantially from the values estimated through indel formation in HEK293T cells (that were typically ≥10%, Figure 1C) perhaps by HEK293T-suppressed innate immune responses, overall robustness of this cell line, and hetero-/eu-chromatization differences between cell types. For instance, the intergenic GSH site *Dep.36* displayed high propensity to genome editing in HEK293T with approximately 30% indels formed (Figure 1C) but yielded the lowest level of GFP^+^ cells after the nucleofection of CD34^+^ HSPCs (Figure 2C).

Although we consulted available ATAC-seq data for CD34^+^ HSPCs^46^ to determine the relative position of EPVs with respect to accessible chromatin regions (Table 1), no evident correlation was observed between editing efficiency and the proximity to reported open chromatin regions (Table S2). An extreme example is the intronic locus *Dep.2* (host gene *KIF6*) which resulted in the highest level of GFP^+^ cells at two days post-nucleofection (3.23% ± 0.86, *p* = 0.8938) despite having 15 kb distance from the nearest reported ATAC peak (Tables 1 and S2). Nevertheless, persistent *eGFP* expression was monitored through differentiated stages of edited *Dep.2* cells (see below), a phenomenon replicated by an additional eight loci, namely, the intergenic loci *Dep.3*, *Dep.22*, and *Ap.102*, and the intronic sites *Dep.55*, *Prot.218*, *Prot.2*, *Prot.181*, and *Dep.1* (host genes: *BCAS4*, *ENPP2*, *PDZRN4*, *MANCR*, and *PAX5*, respectively) (Table S2; Figures 2C and 4A).

The post-editing GFP+ cell viability varied between 75.15% ± 20 live cells (mean ± SD, *p* = 0.0551) after targeting the intronic site *Prot.181* (host gene *MANCR*), and 94.65% ± 3.71 live cells (*p* = 0.9994) for the intergenic site *Dep.13*, as compared to the 98.4% ± 0.92 of the mock editing control (i.e., electroporated cells in absence of any CRISPR-Cas9-ssDNA editing set) (Figure 2D). The proliferation decreased for most of the manipulated CD34^+^ HSPCs within three days following cell sorting compared to control group (6.19-fold ±1.96), excepting for the *AAVS1* (4.43-fold ±1.22, *p* = 0.3294), *Dep.1* (4.56-fold ±1.53, *p* = 0.4331), *Dep.2* (4.21-fold ±0.50, *p* = 0.1373), and *Dep.35* (5.17-fold ±0.18, *p* = 0.9885) editing groups (Figure 2E). Despite this response, large colonies of differentiated cells formed independently of the edited GSH locus demonstrating that neither genome modification nor *eGFP* transgene expression impaired permanently the proliferation capacity of the cells (Figure 4A).

### LT-HSC-like populations are reachable by GSH-targeting

Within the CD34^+^ HSPC cell population, long-term hematopoietic stem cells (LT-HSCs) are those with self-renewal and long-term engraftment potential; therefore, they are considered the most clinically relevant cell group for autologous cell therapy.^53^ In the human, the LT-HSCs are very scarce: estimations report approximately 11,000 HSCs as the reserve of the most primitive cells in bone marrow.^54^ To determine if manipulated cells with HSC features were among the population of CD34^+^ cells in the experiments, we analyzed the GFP^+^ cell fraction to distinguish potential LT-HSCs (CD34^+^/CD90^+^) from the more abundant progenitor cells (CD34^+^/CD90^−^/CD38^+^, CD34^+^/CD90^−^/CD38^−^).^55^ Interestingly, the results suggest that some loci might be accessible in potential LT-HSCs (Figure 2F), including *AAVS1* (10.32%), *Ap.102* (35.97%), *Dep.3* (12.07%), *Dep.28* (2.05%), *Dep.22* (8.56%), *Prot.181* (10.59%), *Dep.2* (8.33%), *Prot.176* (16.67%), *Dep.1* (34.7%), *Prot.2* (11.18%), *Dep.35* (13.22%), *Dep.55* (26.09%), and *Prot.218* (5.6%), making these attractive candidates for future studies (Figure 2F).

### Gene addition is stable in human CD34^+^ HSPCs and can be improved through rAAV6 transduction

To characterize the transgene insertion accuracy into the GSH candidates, genomic DNA from GFP^+^ HSPCs (sorted after 14 days of liquid culture) was analyzed by droplet digital (dd)PCR with locus-specific primer/probe sets spanning the junction region, and an unedited part of the respective locus, thus obtaining the total number of edited and unedited alleles (Figures 2G and S2). Considering that there are two alleles for each GSH per diploid genome, a ratio of 0.5 of edited/total alleles in the sample represents 100% percent of mono-allelic integration, whereas a ratio of 1 is indicative of 100% bi-allelic integration, and ratios in the range of 0.5 to 1 represents partial bi-allelic integration. Thus, the cell population of *AAVS1* edited cells (reference) occurred with an average ratio of 0.65 indicating roughly 40% of bi-allelic integration, whereas intergenic (*Dep.3*, *Dep.13*, *Dep.33*, and *Ap.102*) and intronic GSH (*Dep.1*, *Dep.2*, *Dep.35*, *Prot.2*, *Prot.181*, *Prot.176*, and *Prot.218*) edited cells were almost entirely mono-allelic (Figure 2G). In addition, the transgene was stable for at least 45 days of liquid culture as shown for *AAVS1*, *Dep.2*, and *Dep.3* HSPCs (Figure 2H). No integration events were detected for *Dep.34*, *Dep.36*, *Dep.28*, and *Dep.56* loci and eGFP fluorescence was either very weak or not present (not shown). The ineffective editing of these loci may be attributed to inaccessibility to the editing machinery, unfavorable ssDNA template structure (in the case of the *Dep.56*-template), disruption of vital pathways, or human silencing hub (HUSH)-mediated repression of intronless transgenes,^56^ making them poor candidates for further analysis.

We also analyzed individual colony forming units (CFUs) obtained after 14 days of cell differentiation (Figure 4A) as these represent clones arising from single modified cells. Clonal colonies from three intronic (*Dep.1*, *Dep.2*, and *Dep.55*), or two intergenic GSHs (*Dep.3* and *Ap.102*), and *AAVS1*-edited cells were evaluated for editing (Figure 2G, inset). Bi-allelic integration was observed in for each of the evaluated GSHs, although this was particularly notable on *AAVS1-* and *Dep.1*-derived cells (Figure 2G).

The recent studies in Wiskott-Aldrich, and β-hemoglobinopathy patients, have estimated that as few as 98 corrected HSPCs per 10^6^ infused CD34^+^ cells might engraft in the bone marrow for long term-production of granulocytes.^53^ Given that our experiments were based on relatively small numbers of cells (2.5 × 10^5^ to 1 × 10^6^ CD34^+^ HSPCs per single nucleofection to target one candidate GSH), the results suggest that the scaling-up to clinical readiness (usually >5 × 10^6^ electroporated cells) would increase the population of edited LT-HSCs.^9^^,^^57^ Alternatively, using rAAV6 vectors reportedly substantially increased the editing efficiency. To assess this approach, we combined nucleofection and rAAV6 transduction to deliver homology arm-carrying templates targeting three intergenic (*Ap.102*, *Dep.33*, and *Dep.13*), or two intronic (*Dep1* and *Dep.35*) loci that were inefficiently edited with the nucleofection alone i.e., CRISPR/Cas9-ssDNA templates. Equivalent cell numbers were used among groups (i.e., 2.5 × 10^5^ cells) with *AAVS1* serving as the reference GSH standard. As expected, the percentage of GFP^+^ cells increased dramatically without effecting cell viability (Figure 2I). However, as the rAAV-delivered transgenes tend to persist episomal^58^ using rAAV6 for HSC editing therefore requires additional molecular characterization to determine accurately the integration profiles of the eGFP+ cells (Figures 2I and S1G). Regardless, the results demonstrate that AAV6-mediated gene delivery may increase the editing efficiency of apparently difficult GSH candidates. Notwithstanding, the experiments described hereafter were performed with ssDNA templates.

### Transcriptomes of manipulated HSPCs show no evidence of malignant transformation after gene addition

**Figure 3:**
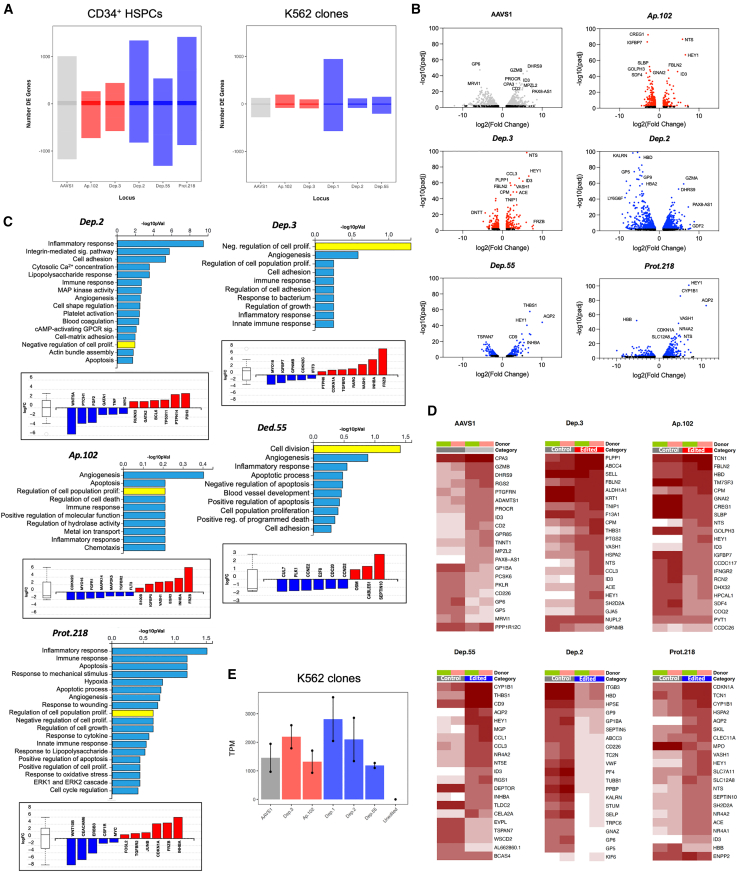
Global gene expression changes following the targeting of GSH candidates show no evidence of malignant transformation of CD34^+^ HSPCs (A) Total DEGs obtained in CD34^+^, or K562 clones, after targeting intergenic (*Dep.3*, *Ap.102*), or intronic (*Dep.1*, *Dep.2*, *Dep.55*, *Prot.218*) GSHs represented as red and blue bars, respectively. *AAVS1* (gray) is used as a reference. Genes consistently upregulated (*n* = 30) and downregulated (*n* = 15) across the experiments are indicated by shading in barplots. The total number of DEGs are lower in clonal K562 cell lines compared to edited primary CD34^+^ HSPCs. (B) Volcano plots of the global transcriptional changes in CD34^+^ cells after editing of *AAVS1* (1008 up-/587 downregulated genes), *Ap.102* (133 up-/363 downregulated genes), *Dep.3* (220 up-/289 downregulated genes), *Dep.2* (1342 up-/821 downregulated genes), *Dep.55* (269 up-/656 downregulated genes), and *Prot.218* (798 up-/436 downregulated genes). Genes with adjusted *p* values <0.01 are highlighted in color, *AAVS1* group is in gray, intergenics in red, and intronics in blue. Highly significant DEGs are identified. (C) GO annotations from edited CD34^+^ cells (*Dep.2*, *Dep.3*, *Ap.102*, and *Prot.218* GSHs). Gene groups with attributes to regulate cell proliferation are especially represented (some highlighted in yellow). Distinctive cancer-related genes, downregulated (blue), or upregulated (red), are plotted at the bottom of the respective chart. (D) Heatmaps with the top 20 significant DEGs per targeted locus. The host genes for intronic GSHs *Dep.55*, *Dep.2*, or *Prot.218* (*BCAS4*, *KIF6*, or *ENPP2*, respectively), or the flanking genes in the case of intergenic GSHs *Dep.3* (*NUPL2*, *GPNMB*), and *Ap.102* (*PVT1*, *CCDC26*), are appended at the bottom demonstrating minimal changes after genome editing. Color scale represents the Log_2_ of normalized counts of *n* = 2 independent donors (pink and green boxes). (E) *eGFP* transcripts per million reads (TPMs) determined in K562 clones to compare the transcriptional capabilities among GSHs.

To determine the extent to which GSH-editing perturbs the transcriptional homeostasis, RNAseq was performed with the bulk of sorted GFP^+^ HSPCs from three intronic (*Dep.2*, *Dep.55*, and *Prot.218*) and two intergenic (*Dep.3* and *Ap.102*) GSHs targeted with ssDNA *eGFP* templates. Differentially expressed genes (DEGs), were determined by comparing to unedited control cells from the same human donor (mock nucleofection). Consequently, the targeting of *AAVS1* resulted in 1,595 DEGs, whereas the intronic GSH candidates *Dep.2*, *Dep.55*, and *Prot.218* (host genes *KIF6*, *BCAS4*, and *ENPP2*, respectively), resulted in 2,163 DEGs, 925 DEGs, and 1,144 DEGs, respectively (Figures 3A and 3B). Perhaps unsurprisingly, the genome editing of intergenic GSH sites, *Dep.3* and *Ap.102*, resulted in fewer DEGs reporting 509 and 496, respectively (Figures 3A and 3B), suggesting that edited intergenic GSH sites tend to perturb the transcriptome to a lesser extent than edited intronic GSH loci.

Similar experiments were conducted with clonally established K562 cells genetically modified into the intronic loci *Dep.1*, *Dep.2*, or *Dep.55* (host genes *PAX5*, *KIF6*, and *BCAS4*, respectively), or the intergenic loci *Dep.3*, and *Ap.102*. RNAseq of unedited K562 clones were used to establish the DEGs. Excepting the *Dep.1* locus, the genome editing of K562 cells resulted in substantially less number of DEGs compared to those obtained in CD34^+^ HSPCs (Figures 3A and S3A). The targeting of intronic loci *Dep.1*, *Dep.2*, or *Dep.55* generated 759, 100, and 179 DEGs, respectively, whereas edited intergenic sites *Dep.3*, or *Ap.102*, produced 94, and 140 DEGs, respectively (Figure 3A). The *AAVS1* reference clone resulted in 204 DEGs. Thus, the data demonstrate that insertion into candidate GSHs induce relatively low transcriptional disturbances, especially from intergenic GSHs. No transcriptional changes were observed in the host genes for intronic GSHs, or in those genes proximal to the insertion sites (Figure S3B). Moreover, the eGFP transgene expression was similar among the clones, indicating equivalent expression conditions from every GSH (Figure 3E).

The Gene Ontology (GO) analysis of the DEGs identified in manipulated CD34^+^ cells revealed no suspicious changes associated with malignant transformation, i.e., no upregulation of oncogenes and down-regulation of tumor suppressor genes. Similar data were obtained with edited K562 cells (Figures 3B, 3C, and S3A). Interestingly, tumor suppressor transcripts were upregulated, including: *CDKN1A* (8.52-fold change, *p* ≤ 10^−8^), *INHBA* (29.1-fold change, *p* ≤ 0.001), and *TP53I11* (2.5-fold change, *p* ≤ 0.01). In addition, we observed downregulation of leukemia-associated proto-oncogenes such as *CCND1/2* (0.46, *p* < 0.05), *CCNE2* (0.38 *p* = 0.0076), *MYC* (0.42, *p* < 0.001), *SRC* (0.3, *p* < 10^−5^), or *MPL* (0.25, *p* < 0.05) (Figures 3C, S4A, and S4B). Finally, targeting the intergenic GSH sites *Dep.3* and *Ap.102* resulted in downregulation of the AML-associated proto-oncogene *FLT3* (0.44, *p* ≤ 0.0014) and overexpression of *INHBA* (18.4-fold change, *p* ≤ 0.005) (Figure 3C). With the exception of *AAVS1* edited cells, all the GSH-targeted cells underwent overexpression of *HEY1* (98.8-fold, *p* < 10^−40^, for *Dep.3*, *Dep.55*, *Ap102*, and *Prot218*), or upregulation of *HES7* (8.9-fold *p* = 0.017 for *Dep.2*), both involved in maintenance of blood precursors (Figure 3D). Overexpression of *FRZB* (74.2, *p* < 0.01), a *β*-catenin pathway-inhibitor, was also detected (Figures 3B and 3D). All the edited GSH groups displayed upregulation of innate immune response-associated genes presumably resulting from nucleofection of ssDNA, CRISPR-Cas9 complex, etc. (Figure 3C). These changes explain the observed *in vitro* phenotype of manipulated CD34^+^ HSPCs, i.e., reduced cell proliferation (Figure 2E), indicating low propensity to malignant transformation at the moment of analysis.

### Edited HSPCs retained multipotency and display either broad or lineage-restricted transgene expression

**Figure 4:**
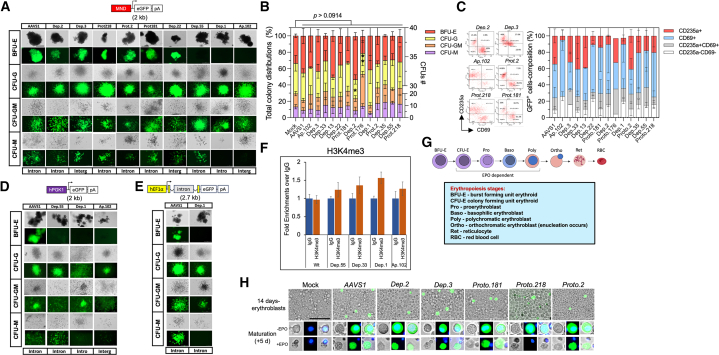
Multipotency of modified CD34^+^ HSPCs is retained after gene addition (A) CFUs per targeted locus after 14 days of differentiation (bright field and epifluorescence micrographs). The GSH locus designation is indicated on the top, the category is at the bottom, and the CFU identity on the left (E, erythroid; G, granulocyte; GM, granulocyte–macrophage and M, macrophage). *AAVS1*, *Dep.2*, *Dep.3*, *Proto.218*, *Proto.2*, and *Prot.181* GSHs evidenced widespread transgene expression, whereas *Dep.55*, *Dep.1*, and *Ap.102* shown expression mostly in CFU-G, CFU-GM, and CFU-M linages. Scale bars, 200 μm (shown in the first image). (B) Total colony distributions per targeted GSH experimental group scored after 14 days of differentiation. The average number of colonies per lineage is indicated on the second *y* axis. Mean ± SD, *n* = 3 independent experiments. Two-way ANOVA followed by Dunnett test ∗*p* < 0.05, ∗∗*p* < 0.01. (C) Erythroid (CD235a^+^) and immune (CD69^+^) composition of the GFP^+^ cell populations through cell flow analysis. Representative scatter plots are shown (left). Mean ± SD, *n* = 2–3 independent experiments. (D) hPGK1 promoter-driven transgene expression from *Dep.55*, *Dep.1*, or *Ap.102* loci. (E). hEF1α promoter-driven transgene from *Dep.1* GSH. (F) CUT&RUN qPCR assay with primers amplifying the MND promoter shows presence of H3K4me3 (activation) on the transgene inserted in *Dep.55*, *Dep.33*, *Dep.1*, or *Ap.102* GSH sites of K562-derived megakaryocytes. Controls of antibodies utilized for immunoprecipitation are available in Figure S6. *n* = 3 experimental replicates. (G) Erythroid maturation stages induced by hEPO. (H) Erythroid maturation from *AAVS1*, *Dep.2*, *Dep.3*, *Prot.181*, *Prot.2*, and *Prot.218* unsorted cultures. Top, 14 days-erythroblasts retain GFP^+^ signal. Scale bars, 100 μm. Bottom, enucleation occurs in presence (+EPO) but not in absence (-EPO) of human EPO. Nuclear counterstaining with Hoechst 33342. Representative pictures of *n* = 2 independent human donors. Scale bars, 100 μm (shown in the first image).

Edited GFP+ HSPCs were cultured in semisolid methylcellulose differentiation medium, and after 14 days no morphological abnormalities were observed among the cell colonies (CFUs) (Figure 4A). HSPCs edited in the reference locus *AAVS1*, and the *Dep.2*, *Dep.3*, *Prot.218*, *Prot.2*, and *Prot.181* loci produced GFP^+^ colonies of each of the major lineages: erythroid (BFU-E), granulocyte (CFU-G), granulocyte-macrophage (CFU-GM) and macrophage lineages (CFU-M) (Figure 4A). In contrast, *Dep.13*, *Dep.33*, *Dep.35*, and *Proto.176*-derived CFUs showed a reduced GFP signal presumably due to transgene downregulation or other undefined causes (not shown). Interestingly, other loci (*Dep.1*, *Dep.55*, and *Ap.102*) displayed *eGFP* expression biased toward CFU-G, CFU-GM, and CFU-M lineages based on visual inspection of the colonies (Figure 4A). Despite dissimilar expression phenotypes, the total colony distributions were similar to unedited control cells (mock) (Figure 4B). Notably, the GSH loci with the highest fluorescent eGFP intensity and unbiased expression corresponded to *AAVS1* (reference locus), *Dep.3*, *Dep.2*, *Dep.55*, and *Prot.218*, which previously showed targeting of potential LT-HSCs (Figure 2F) making these loci attractive candidates for therapeutic applications in human CD34^+^ HSPCs.

To confirm the multipotency of the edited HSPCs, we analyzed the erythroid (CD235a^+^) and myeloid immune (CD69^+^) composition of the GFP^+^ cell populations with *AAVS1* edited cells serving as the reference group.^59^^,^^60^^,^^61^^,^^62^^,^^63^ Erythroid and myeloid immune cells were predominantly detected (Figure 4C), although minor fractions of CD235a^−^CD69^−^ cells (i.e., cells emitting GFP^+^ only), and GFP^+^CD235a^+^CD69^+^ triple labeled cells (i.e., cells with an ambiguous lineage) were also observed. A tendency toward myeloid immune lineage restricted expression was detected from *Dep.1*, and *Ap.102* GSHs, but slightly for the *Dep.55* locus.

These results prompted us to test whether the retroviral components of the MND promoter are related to the lineage controlled regulation, or whether this is a phenomenon unrelated to the promoter and influenced by other events, e.g., epigenetic silencing, chromosomal architecture, etc. Therefore, we evaluated the *eGFP* expression using two cellular promoters: human phosphoglycerate kinase 1 (*hPGK1*) promoter, and human elongation factor 1α (*hEF1α*) promoter. The *hPGK1* promoter-driven transgene was targeted to the *Dep.55*, *Dep.1*, or *Ap.102* locus, finding out eGFP proclivity toward immune lineage expression despite that *hPGK1* is a housekeeping, and weaker promoter, relative to MND (Figure 4D). The *hEF1α* promoter-driven transgene produced the strongest signal from the intronic locus *Dep.1* (host gene *PAX5*), where expression also maintained an immune branch preference (Figure 4E). These data suggest that the endogenous epigenetic array might influence the transgene expression from some GSHs, perhaps following the expression pattern of the host gene in the case of intronic GSHs, case of the *Dep.55* (host *BCAS4*), and *Dep.1* (host *PAX5*) loci, demanding further characterization.

The myeloid immune transgene regulation was explored in edited K562 cells upon targeting of *Dep.1*, *Dep.55*, *Dep.33,* or *Ap.102* GSH. The cells were induced to erythroid (CD235a^+^) or megakaryocytic (CD61^+^) phenotypes by the treatment with erythropoietin (hEPO), or phorbol myristate acetate (PMA), respectively (Figures S5A and S5B). Megakaryocytes were used to model immune cells due to recently described immune roles,^64^ and separated origin from erythrocytes.^65^ Following induction with PMA, the megakaryocytic cells retained strong expression of the *eGFP* (Figure S5C). The presence of either H3K4me3 (activation) or H3K27me3 (repression) in these cells was assessed through CUT&RUN-qPCR (Cleavage Under Targets & Release Using Nuclease). The MND promoter-transgene was enriched with the presence of H3K4me3, but not H3K27me3 when inserted into the assessed GSHs (*Dep.1*, *Dep.55*, *Dep.33*, or *Ap.102*) consistent with a favored transgene expression in immune cell lineages (Figures 4F and S6). The results from hEPO-induction experiments were less conclusive in part due to the low level of differentiated K562 cells obtained after hEPO treatment, such that further characterization will be necessary (Figure S5D).

### Transgene expression persists in terminally differentiated erythroid cells

The final steps of erythroid differentiation involve erythroblast enucleation (Figure 4G). To determine whether the targeting on CD34^+^ HSPCs interfered with erythroid maturation, the *Dep.2*, *Dep.3*, *Prot.218*, *Prot.2*, and *Prot.181* edited groups were exposed to hEPO. Briefly, following the nucleofection of CD34^+^ HSPCs, the cells were expanded during 14 days in erythroblast stimulating conditions (Figure 4H). Next, the erythroblasts were exposed to hEPO for an additional five days of culture after which enucleation was confirmed on GFP^+^ cells, but not in non-hEPO treated cells (Figure 4H). Of note, persistent transgene expression was observed in reticulocytes derived from all analyzed loci except for *Prot.218* in which the transgene expression was silenced soon after exposure to hEPO.

## Discussion

The results of these experiments demonstrated the feasibility of using evolutionary biology and comparative genomic approaches to identify prospective GSHs in the human genome. Of the previously 200 unique EPV insertions identified,^45^ approximately half were mapped to orthologous sites in the human genome indicating that these loci are broadly conserved. In contrast to reported approaches that rely on arbitrary assumptions considered to reduce risks of insertional mutagenesis,^17^^,^^20^^,^^22^ the current study was intended to characterize hematopoiesis-compatible GSH loci.

Potential GSH candidates for gene therapy of blood disorders were identified and characterized *in vitro*, and further *in vivo* evaluation will demonstrate the ability of modified HSPCs for long-term engraftment of bone marrow and reconstitution of blood cell compartments. Ideally, using non-human primate models rather than humanized mice, as the complications associate to malignant clones might not appear for years.^2^^,^^3^ Additionally, early phases of hematopoietic reconstitution are sustained by progenitors and short-term HSCs, until their exhaustion after 2 years^66^ Although the experiments reported here were performed with relatively low numbers of HSPCs (i.e., 2.5 × 10^7^) the results indicate editing of potential LT-HSCs (CD34^+^,CD90^+^).^55^ In clinical applications, approved therapies reportedly utilize ≤2.0–3.0 × 10^7^ autologous CD34^+^ HSPCs per kg of body weight (retroviral or lentivirally transduced) as the cell number for infusion. Therefore, targeting a GSH with sufficient editing efficiency (partially determined by the access to the genomic locus and “quality” of the gRNA) would yield a population of productive stem cells, or CAR-T cells, reducing the apheresis procedures required to isolate the target cells. As the proposed GSHs displayed promising performance, the upscaling for clinical readiness (usually >5 × 10^6^ electroporated cells) might result in increased numbers of engraftable LT-HSCs.

Despite the low editing percentages for some loci (e.g., *Dep.1* or *Ap.102*), the values were enhanced by combined nucleofection/rAAV6 transduction, although the episomal persistence of the rAAV vector genomes must be carefully considered for this approach (Figure 2H). Additionally, most of the GSH loci were compatible with long-term transgene expression in differentiated cells making unnecessary using artificial insulators to bypass the potential transgene silencing as reported elsewhere.^22^^,^^67^^,^^68^ In this regard, a naturally occurring insulator in *AAVS1*^69^ may confer the robust expression often reported from this site^70^ (Figures 2C and 4D). Whether these GSH sites contain natural insulators was not investigated. Nevertheless, the presented approach to identify sites tolerant to gene addition, resulted in a catalog of 102 potential GSHs. The immune specific-GSH regulation might also introduce an additional level of safety, particularly when considering that intergenic GSHs convey less transcriptome disturbances (Figures 3A and 4). Conclusively, potential GSH sites are likely to emerge from the remaining group of mapped loci with prospective therapeutic applications in HSPC and other progenitor cells.

### Limitations of the study

While our approach identified and partially characterized GSH candidates for gene therapy of blood disorders, the heterogeneity of primary CD34^+^ cell populations must be thoroughly considered. The present study was intended to test the gene addition into GSHs from samples coming from genetically variable individuals (as would be expected from an actual clinical trial), and although potential LT-HSCs were reached, the transcriptomic disturbances in actual stem cells must be confirmed through deep sequencing at single-cell level, individual CFUs, or alternatively homogeneous stem cell systems capable of stable expression and prolonged culture (like hESC or iPSCs). Likewise, the engraftment capacity of modified LT-HSCs must be proved preferably in animal models of appropriated lifespan (ideally non-human primates) as complications associated with gene therapy might not appear for years. Even when the GSH loci were predicted not contain open reading frames, it is accepted that evolutionary conserved non-coding sequences might acquire regulatory roles of the gene expression. Thus, additional molecular and cellular analyses are required to fully characterize each locus.

## Resource availability

### Lead contact

Requests for further information and resources should be directed to and will be fulfilled by the lead contact, Robert M. Kotin (robert.kotin@umassmed.edu).

### Materials availability

This study did not generate new unique reagents.

### Data and code availability

• Sequence data generated for this study have been deposited at the National Center for Biotechnology Information.
• Sequence Read Archive: BioProject PRJNA1022496 is publicly available as of the date of publication.
• Any additional information required to reanalyze the data reported in this paper is available from the lead contact upon request.

## Acknowledgments

This work was supported in part by funding from the Association Monégasque Contre les Myopathies (R.M.K.), and the 10.13039/100000865Bill & Melinda Gates Foundation (OPP1202116 to R.M.K.). Cell sorting experiments were supported by 10.13039/100000002NIH S10OD028576. We are grateful to Prof. Oliver J. Rando and his lab for providing access to the NextSeq 500/550 platform for sequencing our libraries.

## Author contributions

R.M.K., conceptualization; R.M.K, M.Q.-R., and K.P.M., methodology; M.Q.-R., M.A.C., and K.M.P., investigation; M.Q.-R., writing – original draft; M.Q.-R., M.A.C., K.M.P., R.G., and R.M.K., writing – review & editing; R.M.K., funding acquisition; M.A.C., K.M.P., R.G., and R.M.K., resources; R.M.K., supervision.

## Declaration of interests

This article presents results that are covered by intellectual property co-owned by the University of Massachusetts Chan Medical School and Synteny Therapeutics. R.M.K. is co-founder of Synteny Therapeutics, Inc.

## STAR★Methods

### Key resources table

**Table:** 

REAGENT or RESOURCE	SOURCE	IDENTIFIER
**Antibodies**		

Anti-human CD34	BioLegend	Cat# 343625; RRID: AB_2632726
Anti-human CD69	BioLegend	Cat# 310909; RRID; AB_314844
Anti-human CD235a	BioLegend	Cat# 349105; RRID; AB_10641707
Anti-human CD90 (Thy1)	BioLegend	Cat# 328114; RRID: AB_893431
Anti-human CD38	BioLegend	Cat# 303506; RRID: AB_314358
Anti-human CD61	BioLegend	Cat# 336405; RRID: AB_1227583
IgG control antibody	EpiCypher	Cat# 13-0042; RRID: AB_2923178
Anti-H3K4me3	EpiCypher	Cat# 13-0041; RRID: AB_3076423
Anti H3k27me3	Activemotif	Cat# 39055; RRID; AB_2561020

**Bacterial and virus strains**		

AAV6_Ap.102	This report	N/A
AAV6_Dep.33	This report	N/A
AAV6_Dep13	This report	N/A
AAV6_Dep.1	This report	N/A
AAV6_Dep.35	This report	N/A

**Biological samples**		

Healthy donor CD34^+^ cells from bone marrow	Stem Cell Technologies	Cat#

**Chemicals, peptides, and recombinant proteins**		

ArchiTect™ Cas9 nuclease	Stem Cell Technologies	Cat# 76004
SFEM II medium	Stem Cell Technologies	Cat# 09655
CD34^+^ Expansion Supp	Stem Cell Technologies	Cat# 02691
UM729	Stem Cell Technologies	Cat# 72332
Erythroid Expansion Supp	Stem Cell Technologies	Cat# 02692
Erythropoietin	Stem Cell Technologies	Cat# 78007
MethoCult	Stem Cell Technologies	Cat# H4435
Trizol	Thermo Fisher Scientific	Cat#15596026

**Critical commercial assays**		

Guide-it Long ssDNA Production System v2	Takara Bio	Cat# 632666
SMARTer-seq Total RNA-Seq Pico input	Takara	Cat# 634357
SMARTer RNA Unique Dual Index Primers	Takara	Cat# 634756
AMpure XP beads	Beckman coulter	Cat# A63880
EpiCypher CUTANA™ ChIC/CUT&RUN kit	EpiCypher	Cat# 14-1048
P3 Primary Cell 4D-Nucleofector™ X Kit S	Lonza	Cat# V4XP-3032

**Deposited data**		

Sequence data generated have been deposited in the National Center for Biotechnology Information Sequence Read Archive	This report	BioProject PRJNA1022496

**Experimental models: Cell lines**		

HEK293T	ATCC	CRL-3216
K562	ATCC	CCL-243

**Oligonucleotides**		

Dep.3 gRNA AGGCTTCGGCTTCCGCAACG	Integrated DNA Technologies, Inc.	This report
Ap.102 gRNA CCATTATTCATGGAGCACGC	Integrated DNA Technologies, Inc.	This report
Dep.28 gRNA TACTAATGCAGGTGTACATA	Integrated DNA Technologies, Inc.	This report
AAVS1 gRNA GGGCCACTAGGGACAGGAT	Integrated DNA Technologies, Inc.	Mali et al.^47^
Proto.2 gRNA ATATAATTACTTAAGGCTGT	Integrated DNA Technologies, Inc.	This report
Dep.1 gRNA GAGGCACCGTGATGCCGCAG	Integrated DNA Technologies, Inc.	This report
Proto.218 gRNA TCCTCCGAACTCCACTAGAA	Integrated DNA Technologies, Inc.	This report
Proto.176 gRNA GTACCTATCTGCCACAGTAC	Integrated DNA Technologies, Inc.	This report
Dep.2 gRNA GTATACAATAATATGAAGAG	Integrated DNA Technologies, Inc.	This report
Dep.55 gRNA GGGAAGATCACCTGGGCTCT	Integrated DNA Technologies, Inc.	This report
Dep.33 gRNA AACACCTAGCATTCACATAA	Integrated DNA Technologies, Inc.	This report
Dep.34 gRNA GTATGATGCAACTTGGTCCG	Integrated DNA Technologies, Inc.	This report
Dep.36 gRNA AGTAACCAAGGATTCCTGCT	Integrated DNA Technologies, Inc.	This report
Dep.22 gRNA CTTCTAACAAACCAAACATG	Integrated DNA Technologies, Inc.	This report
Dep.13 gRNA TCCCTGGTCCACCTTAAGGC	Integrated DNA Technologies, Inc.	This report
Dep.56 gRNA GCCCAGTGTAATCAAAGACA	Integrated DNA Technologies, Inc.	This report
Dep.35 gRNA GCACATACACTCATGGGTAG	Integrated DNA Technologies, Inc.	This report
Proto.181 gRNA ACTATACTCCTTGCTCCCAC	Integrated DNA Technologies, Inc.	This report
AAVS1 F (ssDNA synthesis) ATTCGGGTCACCTCTCAC	Integrated DNA Technologies, Inc.	
AAVS1 R (ssDNA synthesis) ACGGCCGCGTCAGAGCA	Integrated DNA Technologies, Inc.	5Phos
Dep.34 F (ssDNA synthesis) ATCACTGAAATCATAAATAGGATTTAGAAT	Integrated DNA Technologies, Inc.	
Dep.34 R (ssDNA synthesis) TGCACTTGAACTGAAAGTTATTC	Integrated DNA Technologies, Inc.	5Phos
Dep.36 F (ssDNA synthesis) ATTCAGTTGAACTCAGATAACTCGA	Integrated DNA Technologies, Inc.	
Dep.36 R (ssDNA synthesis) TCAGTTTGTAAGTTTTAGATAGTTCC	Integrated DNA Technologies, Inc.	5Phos
Ap.102 F (ssDNA synthesis) GCTCCTTTTCCCCAGTGG	Integrated DNA Technologies, Inc.	
Ap.102 R (ssDNA synthesis) TGCTTCCAAGTTGCCTAAAAT	Integrated DNA Technologies, Inc.	5Phos
Dep.3 F (ssDNA synthesis) ACGCTAATGTCTTACTGAAATATAC	Integrated DNA Technologies, Inc.	
Dep.3 R (ssDNA synthesis) CTGGGTCTGGGAACACCAC	Integrated DNA Technologies, Inc.	5Phos
Dep.33 F (ssDNA synthesis) TGAGTTTGATGGAGAAAGATGGT	Integrated DNA Technologies, Inc.	
Dep.33 R (ssDNA synthesis) CTGCAGTCCACTAATGAATTAATAG	Integrated DNA Technologies, Inc.	5Phos
Dep.13 F (ssDNA synthesis) ACAGTTCCACGTGGCTGGG	Integrated DNA Technologies, Inc.	
Dep.13 R (ssDNA synthesis) CAGGAAGCATCATTGTCTCC	Integrated DNA Technologies, Inc.	5Phos
Dep.28 F (ssDNA synthesis) GGTAAGCCAGAGCTTTTTCTACT	Integrated DNA Technologies, Inc.	
Dep.28 R (ssDNA synthesis) TACACTGTTGTTGTTGTTGTTTTTTTT	Integrated DNA Technologies, Inc.	5Phos
Dep.22 F (ssDNA synthesis) CCAGCCAGCCTTAAAATTAGAT	Integrated DNA Technologies, Inc.	
Dep.22 R (ssDNA synthesis) TGCAACTAGGTATTTAAACTCAATTCC	Integrated DNA Technologies, Inc.	5Phos
Proto.181 F (ssDNA synthesis) AAATTTATTTTTTATCTGTTGTGAACTAGAGATC	Integrated DNA Technologies, Inc.	
Proto.181 R (ssDNA synthesis) TTCCAGCCTCTAGAACTATGAG	Integrated DNA Technologies, Inc.	5Phos
Dep.2 F (ssDNA synthesis) TCCTGATGGTGCCACACGG	Integrated DNA Technologies, Inc.	
Dep. R (ssDNA synthesis) CAATGCACTCAGCCCCATG	Integrated DNA Technologies, Inc.	5Phos
Prot.176 F (ssDNA synthesis) AAACAAATTTTGTAATTCATGCATC	Integrated DNA Technologies, Inc.	
Prot.176 R (ssDNA synthesis) TCCTCATATGAAAATTTCTGCAAAT	Integrated DNA Technologies, Inc.	5Phos
Dep.1 F (ssDNA synthesis) AGAAAAACCGAGAGAGAGGG	Integrated DNA Technologies, Inc.	
Dep.1 R (ssDNA synthesis) GATTAAACTTGGACCAGTGTTGTTC	Integrated DNA Technologies, Inc.	5Phos
Prot.2 F (ssDNA synthesis) ACCACTACATGATAAAAAGAAAATAAGA	Integrated DNA Technologies, Inc.	
Prot.2 R (ssDNA synthesis) CCACACCTTAAAATACAAGAAGGAG	Integrated DNA Technologies, Inc.	5Phos
Dep.35 F (ssDNA synthesis) GACAGGAAATTTATTAGACAGTTTACAGC	Integrated DNA Technologies, Inc.	
Dep.35 R (ssDNA synthesis) TTCTGGTGCACTAAGATTTTAACT	Integrated DNA Technologies, Inc.	5Phos
Dep.55 F (ssDNA synthesis) TTCTCCTCAGCCAGCCAA	Integrated DNA Technologies, Inc.	
Dep.55 R (ssDNA synthesis) GCCTCGGTATGTGCCAAG	Integrated DNA Technologies, Inc.	5Phos
Prot.218 F (ssDNA synthesis) GGGCAACTTAAATTGAATCAGCT	Integrated DNA Technologies, Inc.	
Prot.218 R (ssDNA synthesis) CCCCGAATCCACTGAGGGAA	Integrated DNA Technologies, Inc.	5Phos
Dep.56 F (ssDNA synthesis) GTCAGTGTACAGTCAACAGCA	Integrated DNA Technologies, Inc.	
Dep.56 (ssDNA synthesis) CTCAGCCTCCCAAAGTGC	Integrated DNA Technologies, Inc.	5Phos

**Software and algorithms**		

CHOPCHOP	University of Bergen	chopchop.cbu.uib.no/
CRISTA	Tel Aviv University	crista.tau.ac.il/
CRISPOR	University of California	crispor.tefor.net/
iPathwayGuide	Advaita Bioinformatics	advaitabio.com
Integrative Genomics Viewer (IGV)	Broad Institute	software.broadinstitute.org/software/igv/
R studio	Posit	cran.r-project.org/

### Experimental model and study participant details

#### Bone marrow CD34^+^ HSPCs from adult healthy donors

Human Bone Marrow CD34^+^ Cells were purchased to Stem Cell Technologies (Catalog #70002) considering adult donors between the ages of 20 and 35 years old, man and women healthy donors, all ethnicities.

### Method details

#### Homology ascertainment relative to human genome

EPV positions were mapped to the orthologous human loci using Ensembl for comparative genomic alignments (GRCh38.p13) (Ensembl 2023). However, since the host species genome sequences accessible via Ensembl may be from different assembly versions than the reported EPV loci sequences the EPV coordinates within host genomes were acquired through BLASTN or BLAT analysis tool within the Ensembl suite. Then, using the Comparative Genomics options in Ensembl, the aligned host species and multiple mammalian genomes were analyzed to ascertain overall synteny and collinearity. Thus, the corresponding human genomic locations were determined. As some EPV host species were not available in Ensembl, BLASTN and EPV genomic flanking sequence data were used to locate the EPV integration site in closely related host species genomes that may or may not contain an EPV ortholog. Gaps in the host/human sequence alignment surrounding the EPV elements introduced another level of ambiguity in identifying sites in which the element may have been inserted.

#### Selection of human genome loci to be evaluated as GSH

We focused on EPV loci that were mapped with a high degree of confidence on the human genome. As the experimental portion of this effort involved editing human primary CD34^+^ HSPCs, we evaluated the accessibility of chromatin for EPVs in this cell type. CD34^+^ HSPC data were retrieved from the Gene Expression Omnibus under accession GSE74912. ATACseq data were accessed with DolphinNext (Corces et al.^46^) and chromatin accessibility was estimated through peak-calling with MACS2.

#### Cell culture and transfection

Human embryonic kidney (HEK)-293T cells (ATCC CRL-3216) were cultured in 10% FBS DMEM (Gibco 11965-092), supplemented with penicillin (50 U per mL) and streptomycin (0.1 mg per mL) (Sigma P4458), at 37°C in 5% CO2 and saturating humidity. Transfection experiments on HEK-293T cells were performed in 6-well plates (Corning, NY). Briefly, one day prior to transfections, 3.5x10^5^ cells were seeded per well. Then cells were transfected with pX330 plasmid (2.5μg) using Lipofectamine 3000 (Invitrogen) following the manufacturer’s instructions. Forty-eight hours post-transfection genomic DNA (gDNA) was isolated with DNeasy Blood & Tissue Kit (Qiagen), and following concentration measurement, an aliquot of gDNA was used for PCR amplification of the targeted loci using locus specific primers to determine editing efficiency and specificity.

Human bone marrow-derived CD34^+^ stem and progenitor cells (HSPCs), obtained from healthy adult donors, were purchased from Stem Cell Technologies (SCT, Cambridge, MA 02142). Briefly, cryopreserved HSPCs were thawed and pre-stimulated for 48 h in StemSpanTM SFEM II medium (SCT 09655) with Expansion supplement (SCT 02691) and 1 μM UM729 (SCT 72332), and incubated at 37°C in 5% CO_2_ and saturating humidity atmosphere. Following electroporation, the CD34^+^ HSPCs were returned to the same incubation conditions for additional 72 h before further downstream use.

Erythroleukemia K562 (ATCC CCL-243) cells were maintained in 10% Iscove’s modified Dulbecco’s medium(IMDM) (10-016-CV, Corning Life Sciences) supplemented with penicillin (50 U per mL) and streptomycin (0.1 mg per mL) (Sigma P4458), at 37°C, 5% CO2 and saturating humidity. Following electroporation, the cells were returned to the growth medium and incubated for 5 days before cell sorting. Sorted cells expressing *eGFP* were expanded under similar conditions for use in subsequent experiments. Single cell clones were obtained by limiting dilution in 96-well plates and screened for *eGFP* expression, and targeted integration.

#### Guide RNAs for CRISPR/Cas9 gene editing

Single guide RNAs (gRNAs) were simultaneously engineered with CHOPCHOP, CRISPOR and CRISTA designing tools. Three to five highly scored gRNAs with minimal off-target sites that were common to the three gRNA design programs, were chosen per candidate GSH locus. Synthetic oligonucleotides incorporating the *gRNA* sequences were ligated into pX330 plasmid (Addgene, Watertown, MA 02472) to co-express *Cas9* and *gRNA*. Indel formation of the target site was used as an indicator for editing efficiency in HEK-293 cells. Forty-eight hours post-transfection into HEK293T cells, genomic DNA was isolated (Qiagen), and PCR amplified into 600 bp products (Takara). Indel frequencies were determined through Sanger data-decomposition using the TIDE analysis tool (tide.nki.nl/). Indel efficiencies were compared to that provided for a widely reported *AAVS1* gRNA. Thus, the most efficient gRNA per locus was selected for downstream experiments in CD34^+^ HSPCs. Next, a chemically synthesized version of each gRNA, modified at both termini with 2’-O-methyl-3’-phosphorothioate, was purchased as a single guide molecule from IDT (Integrated DNA Technologies, IA) and complexed with ArchiTect™ Cas9 nuclease (Stem Cell Technologies) for editing experiments in primary human CD34^+^ HSPCs.

#### Single-stranded DNA template preparations

Single-stranded DNA donor templates (ssDNA) were synthesized from plasmids templates carrying 300 bp homology arms appropriated for each GSH site and carrying a common *eGFP* expressing cassette. Briefly, an *eGFP* expression cassette regulated by the MND promoter and rabbit β-globin poly(A) signal was constructed by conventional molecular techniques. Homology arms were obtained by PCR from HEK-293T genomic DNA such a plasmid template was generated per candidate GSH site through Gibson assembly (New England Biolabs) with fragments synthesized with Q5 High Fidelity Taq polymerase (New England Biolabs). Next, 5’-phosphorylated PCR primers were used to amplify 2.0 kb eGFP expression cassettes flanked with 300 bp homology arms. To produce the single-stranded template, the phosphorylated reverse strand was digested by sequential strandase-treatment (Takara 632666), and subsequently the product was column-purified and resuspended in nucleases-free water (Gibco). Plasmid templates were eliminated from the preparations by DpnI treatment (NEB R0176) prior to the ssDNA synthesis. For experiments involving the PGK promoter, the sequence was cloned again from 293T genomic DNA and inserted into the plasmid templates upon removal of MND promoter.

#### Electroporation

Prior to nucleofection of CD34^+^ HSPCs, ArchiTect™ Cas9 nuclease (SCT) and the corresponding gRNA were combined (1:2.5 molar ratio) to form a ribonucleoprotein complex at 25°C for 20 min and placed at 4°C until used for nucleofection reactions. In parallel, CD34^+^ HSPCs pre-stimulated for two days were washed in 50 mL PBS at 37°C and resuspended in P3 nucleofector solution at RT (Lonza, V4XP-3032). Approximately 2.5x10^5^ CD34^+^ HSPCs were mixed with gRNA:Cas9 complexes and 3 μg ssDNA donor template (20 μL) in nucleofector strip format (Lonza). Electroporation was performed in Unit X, Lonza 4D nucleofector, using the DZ-100 program. After nucleofection, cells were incubated for 10 min at room temperature before adding the StemSpan™ SFEM II medium with Expansion supplement and 1 μM UM729 for a 2 day-recovery time. After the recovery period, the cells were sorted by flow cytometry and GFP^+^ cells were collected. Similarly, erythroleukemia K562 cells were washed in 50 mL PBS at RT and approximately 1x10^6^ were resuspended in SF solution (Lonza V4XC-2032) and electroporated in presence of pre-assembled Cas9:gRNA complexes plus 12 μg ssDNA (100 μL format) in a Lonza 4D-nucleofector using the FF-120 program.

#### AAV6 transduction

Recombinant adeno-associated virus type 6 (rAAV6) vectors were obtained from the University of Massachusetts Chan Medical School Viral Vector Core facility. Cryopreserved CD34^+^ HSPCs were thawed, transferred to tissue culture plates, and incubated in StemSpan^TM^ SFEM II medium for a 2 days pre-stimulation period under conditions described above. Following pre-stimulation treatment, 2.5x10^5^ CD34^+^ cells were electroporated in the presence of Cas9:gRNA complexes, followed by a recovery period of 15 min at RT. Next, cells were collected and transduced with rAAV6 at a multiplicity of infection (MOI) of 200,000-300,000 gc/cell (genome copies) in a 15 mL conical tube (Corning). Cells were transferred to a 6-well culture plate (Corning) at a density of 1x10^5^ cells/mL and incubated for 2 days to recover from the treatment before downstream analysis.

#### Colony forming unit assay (CFU)

Between 300 and 1500 sorted GFP^+^ cells were seeded in 1 mL methylcellulose hematopoietic progenitor cell differentiation medium (MethoCult, SCT H4435) and transferred to 6-well SmartDish plates (SCT 27371). Differentiation was induced during 14 day incubation at 37°C, 5% CO_2_ and saturating humidity before scoring the Colony Forming Units (CFUs) by brightfield microscopic inspection (Axiovert 135 microscope, Zeiss) and fluorescent images were obtained with fluorescent microscopy (LionHeart FX,BioTek). After colony scoring, the entire plate of cells were recovered from methylcellulose by diluting in PBS at room temperature, next washed and resuspended in ice cold 1% FBS in PBS before the staining for flow cytometry analysis as described above.

#### Flow cytometry

After recovering for 2 days, nucleofected CD34^+^ HSPCs were washed once in PBS and resuspended in ice cold phenol red- free-IMDM (Gibco) supplemented with 1% FBS (Gibco) and processed using fluorescence activated cell sorting (FACS). Non-transfected HSPC samples were used for gating GFP^+^ signals. Propidium iodide (Sigma) was included for viability determinations then, GFP^+^ cells were collected and cultured for 3 additional days in StemSpan^TM^ SFEM II, CD34^+^ Expansion supplement and 1 μM UM729. The fold expansion was determined by dividing the number of cells after three days of culture by the number of GFP^+^ cells seeded immediately after sorting. Thus, the total timeline for nucleofection experiments starting with cryopreserved, immature CD34^+^ HSPCs spanned 7 days. For the analysis of immature populations (LT-HSC and MPPs), cells were cultured for five days post-nucleofection, washed in PBS and then resuspended in ice cold PBS supplemented with 1% FBS. Subsequently, cells were incubated in presence of TruStain FcX^TM^ (BioLegend) before staining with antibodies against CD34 (BioLegend 343625), CD90 (BioLegend 328114) and CD38 (BioLegend 303506). Samples were fixed in ice cold 2% paraformaldehyde (PFA) for 10 min, washed and resuspended in phenol red free-IMDM. The GFP^+^/CD34^+^ population was gated to explore the modified immature cell populations. For cell lineage determinations (erythroid and immune), cells were recovered by diluting the methylcellulose in PBS and resuspending the cells in ice cold 1% FBS in PBS. The Fc receptors were blocked with Human TruStain FcX^TM^ (BioLegend) before staining with antibodies against CD235a and CD69 for 20 min at 4°C in the dark. All antibodies used in this work can be consulted in Table S4. Following the incubation, the cells were washed in ice cold PBS and resuspended in ice cold 2% PFA for 10 min, washed once again with ice cold PBS, and finally resuspended in 1% FBS phenol red-free IMDM (Gibco) before the analysis. For experiments with differentiated K562 cells, the cells were stained with antibodies against CD235a (erythroblastic) or CD61 (megakaryocytes) surface markers as described above except that fixation with 2% PFA was omitted as the samples were processed immediately for CUT&RUN epigenetic modification analysis.

#### Erythroid differentiation

Nucleofected CD34^+^ HSPCs were maintained in SFEM II cell culture medium with Erythroid supplement (SCT) to induce erythroblast expansion during 14 day incubation. Medium changes were performed at days 7 and 11 of culture. At day 14, cells were transferred into SFEM II supplemented with 3 U/mL hEPO (SCT) and 3% AB human serum (Sigma) for an additional 5 days of culture. Cell nuclei were stained with Hoechst 33342 (ThermoFisher) prior to microscopy imaging.

#### Erythroblastic and megakaryocyte induction of K562 cells

K562 cells were induced to erythroblastic cells by exposure to 3U/mL hEPO (SCT 78007). Megakaryocytic cells were obtained with 80 nM phorbol 12-myristate 13-acetate (Invivogene). Cellular phenotypes were identified by cell surface markers using anti-CD235a antibody (BioLegend 349105) for erythroblastic and anti-CD61 antibody (BioLegend 336406) for megakaryocytic cells and flow cytometry. The sorted cells were immediately processed for CUT&RUN experiments.

#### Droplet digital PCR

The integration efficiency was determined by ddPCR of genomic DNA extracted from sorted GFP^+^ cells of 14 days post-nucleofection. Briefly, genomic DNA isolated from the cells (Qiagen) was amplified by ddPCR in the presence of a primer/FAM probe set specific for the integrated transgene and a primer/HEX probe set specific for a reference region within the same unaltered locus (find the primers and probes in the Tables S3–S5). Reaction mixtures consisted of 1x ddPCR Supermix for probes (No dUTP) (Bio-Rad), primer/probe mixtures (900 nM primer/250 nM probe) and up to 60 ng gDNA per reaction. The temperature setting was: initial denaturation at 95°C for 10 min (ramp rate of 2°C/sec), followed by 60 cycles of denaturation at 94°C for 30 sec (ramp rate of 2°C/sec), annealing at 55°C for 1 min (ramp rate of 1°C/sec), and 72°C extension for 2 min (ramp rate of 1°C/sec), and a terminal denaturation step at 98°C for 10 min, followed by cooling at 4°C. Droplet Reader and Quantasoft analysis software (Bio-Rad) were used to analyze the fluorescence using 1D and 2D plots for thresholding FAM and HEX positive droplets. Ratios of FAM to HEX were used to calculate the abundance of edited alleles.

#### RNA-seq

Sorted GFP^+^ HSPCs were cultured for three days in StemSpan^TM^ SFEM II medium (SCT) before being washed in PBS. Subsequently, the cells were mixed with five volumes of RNA*later* solution (Thermo Fisher AM7020) and stored at -80°C until processing for RNA-seq analysis. For library preparation, the mRNA fraction was prepared by poly(A)+ selection (Illumina). Libraries were sequenced as paired end 150 bp reads on an Illumina HiSeq 3000 with a sequencing depth of approximately 20-30 million per sample. Quality control metrics were confirmed in FASTQ files through FastQC (bioinformatics.babraham.ac.uk/projects/fastqc/). Adapter sequences were removed from the reads and mapped to the *Homo sapiens* reference genome (GRCh38) available on ENSEMBL using STAR aligner v.2.5.2b. Differential expression analysis was performed by analyzing un-normalized read counts using DESeq2 and applying “donor” as a cofactor. Transcripts with an adjusted p-value < 0.05 and absolute log_2_ fold change > 1 were designated differentially expressed genes (DEGs) as d. Heatmaps with log_2_ normalized read counts were generated with R Studio, clustering genes according to their regulation changes. Gene ontology (GO) analysis was performed with the iPathwayGuide platform of Advaita Bioinformatics (advaitabio.com).

For RNAseq experiments from stable K562 cell lines, two clonally established lines were generated per targeted GSH, and normalized with unedited control cells, also clonally established. All groups were in the same number of culture passage at the moment of library preparations (passage 13) and maintained the same cell confluence. Then, total RNA was extracted using TRIzol reagent (Thermo Fisher Scientific). The quality and integrity of the extracted RNA were assessed using a Fragment Analyzer. Only samples with an RNA Quality Number (RQN) ≥ 8 were selected for further processing. For library preparation, 10 ng of total RNA was used as input for the SMARTer-seq Total RNA-Seq Pico input (Takara). The RNA subjected to chemical fragmentation at 85°C for 4 minutes to achieve a median insert size of ∼180 bp. The first-strand cDNA synthesis was performed using the SMART TSO Mix, which incorporates unique molecular identifiers (UMIs) to mitigate PCR duplicates and improve quantification accuracy Ribosomal cDNA was selectively depleted using the ZapR v2 enzyme in conjunction with mammalian-specific R-Probes v2, ensuring efficient removal of rRNA-derived sequences. Libraries were amplified with SMARTer RNA Unique Dual Index Primers (Takara) and purified with AMpure XP beads (Beckman coulter). The final libraries were paired-end sequenced on the Illumina NextSeq 500/550 platform. Differential expression analysis was performed by analyzing un-normalized read counts using DESeq2. To determine the number of *eGFP* transcripts the DNA coding sequence of human-optimized *GFP* was appended to the transcript files,^71^ and an index prepared for use with Salmon.^72^ Transcripts were quantified in terms of Transcripts Per Million (TPM) by using the quant option of Salmon.

#### CUT&RUN assay

Chromatin samples were processed using the EpiCypher CUTANA™ ChIC/CUT&RUN kit (EpiCypher 14-1048), according to the manufacturer’s protocol. Briefly, approximately 500,000 cells per sample were collected by centrifugation, resuspended in PBS, then re-centrifuged and then coupled to activated Concanavalin A beads. The cells were incubated with antibodies overnight at 4°C. Approximately 0.5ug of an antibody was used per sample, including IgG negative control antibody (EpiCypher 13-0042), H3K4me3 (EpiCypher 13-0041), and H3K27me3 (Active motif 39055). For permeabilization, 0.01% of digitonin was used in the buffers. Unbound antibody was removed by washing 2x, the cells were incubated with protein A and protein G - micrococcal nuclease fusion (pAG-MNase) to recover the histone - DNA complex. MNase was activated by adding 1mM CaCl2 and incubated at 4°C for 2 hours, releasing the chromatin immune complexes into the supernatant. The MNase reaction was terminated using a stop buffer master mix and to remove RNA from the sample. The released DNA fragments were recovered and size selected using SPRIselect beads and eluted in 15ul of elution buffer for subsequent qPCR analysis (Table S6). Genomic DNA extraction was extracted from the input sample (1% of total cells) and the enrichment of immunoprecipitated DNA was calculated using percent input method formula - 100∗2ˆ(Adjusted Input – CT (IP). The final values were plotted as fold enrichments over IgG negative control.

### Quantification and statistical analysis

All the experiments with primary human CD34^+^ cells, or cell lines when indicated, were independently performed at least three times for statistical analysis. If less than 3 experiments were performed no statistics was applied. The *n* size (i.e., number of independent experiments) is indicated in the figure legends. CD34^+^ cells were harvested from different anonymous, healthy bone marrow adult donors and were used for experiment just once, thus *n* equals the number of donors in those experiments. Data represents mean ± SD. Statistical analyses were performed by ANOVA with Dunnett test post hoc. Significant and non-significant *p* values are indicated on the Figures and the main text. Results were considered significant when *p* < 0.05. When less than 3 experiments were performed for descriptive results, an opportune mention is indicated on the figure legends.

## Supplemental information

Document S1. Figures S1–S6 and Tables S2–S6

Table S1. EPVs mapped into the human genome, coordinates, and nomenclature

## References

[1] FDA (2023). https://www.fda.gov/vaccines-blood-biologics/safety-availability-biologics/fda-investigating-serious-risk-t-cell-malignancy-following-bcma-directed-or-cd19-directed-autologous.

[2] Cesana D., Cicalese M.P., Calabria A., Merli P., Caruso R., Volpin M., Rudilosso L., Migliavacca M., Barzaghi F., Fossati C. (2024). A case of T-cell acute lymphoblastic leukemia in retroviral gene therapy for ADA-SCID. Nat. Commun..

[3] Duncan C.N., Bledsoe J.R., Grzywacz B., Beckman A., Bonner M., Eichler F.S., Kühl J.S., Harris M.H., Slauson S., Colvin R.A. (2024). Hematologic Cancer after Gene Therapy for Cerebral Adrenoleukodystrophy. N. Engl. J. Med..

[4] Stadtmauer E.A., Fraietta J.A., Davis M.M., Cohen A.D., Weber K.L., Lancaster E., Mangan P.A., Kulikovskaya I., Gupta M., Chen F. (2020). CRISPR-engineered T cells in patients with refractory cancer. Science.

[5] Boulad F., Maggio A., Wang X., Moi P., Acuto S., Kogel F., Takpradit C., Prockop S., Mansilla-Soto J., Cabriolu A. (2022). Lentiviral globin gene therapy with reduced-intensity conditioning in adults with beta-thalassemia: a phase 1 trial. Nat. Med..

[6] Spencer Chapman M., Cull A.H., Ciuculescu M.F., Esrick E.B., Mitchell E., Jung H., O'Neill L., Roberts K., Fabre M.A., Williams N. (2023). Clonal selection of hematopoietic stem cells after gene therapy for sickle cell disease. Nat. Med..

[7] Gong J., Chung T.H., Zheng J., Zheng H., Chang L.J. (2021). Transduction of modified factor VIII gene improves lentiviral gene therapy efficacy for hemophilia A. J. Biol. Chem..

[8] Kohn D.B., Booth C., Kang E.M., Pai S.Y., Shaw K.L., Santilli G., Armant M., Buckland K.F., Choi U., De Ravin S.S. (2020). Lentiviral gene therapy for X-linked chronic granulomatous disease. Nat. Med..

[9] Rai R., Romito M., Rivers E., Turchiano G., Blattner G., Vetharoy W., Ladon D., Andrieux G., Zhang F., Zinicola M. (2020). Targeted gene correction of human hematopoietic stem cells for the treatment of Wiskott - Aldrich Syndrome. Nat. Commun..

[10] Rio P., Navarro S., Wang W., Sánchez-Domínguez R., Pujol R.M., Segovia J.C., Bogliolo M., Merino E., Wu N., Salgado R., Lamana M.L. (2019). Successful engraftment of gene-corrected hematopoietic stem cells in non-conditioned patients with Fanconi anemia. Nat Med.

[11] Kotin R.M., Linden R.M., Berns K.I. (1992). Characterization of a preferred site on human chromosome 19q for integration of adeno-associated virus DNA by non-homologous recombination. EMBO J..

[12] Kotin R.M., Siniscalco M., Samulski R.J., Zhu X.D., Hunter L., Laughlin C.A., McLaughlin S., Muzyczka N., Rocchi M., Berns K.I. (1990). Site-specific integration by adeno-associated virus. Proc. Natl. Acad. Sci. USA.

[13] Gomez-Ospina N., Scharenberg S.G., Mostrel N., Bak R.O., Mantri S., Quadros R.M., Gurumurthy C.B., Lee C., Bao G., Suarez C.J. (2019). Human genome-edited hematopoietic stem cells phenotypically correct Mucopolysaccharidosis type I. Nat. Commun..

[14] Scharenberg S.G., Poletto E., Lucot K.L., Colella P., Sheikali A., Montine T.J., Porteus M.H., Gomez-Ospina N. (2020). Engineering monocyte/macrophage-specific glucocerebrosidase expression in human hematopoietic stem cells using genome editing. Nat. Commun..

[15] Irion S., Luche H., Gadue P., Fehling H.J., Kennedy M., Keller G. (2007). Identification and targeting of the ROSA26 locus in human embryonic stem cells. Nat. Biotechnol..

[16] Zambrowicz B.P., Imamoto A., Fiering S., Herzenberg L.A., Kerr W.G., Soriano P. (1997). Disruption of overlapping transcripts in the ROSA beta geo 26 gene trap strain leads to widespread expression of beta-galactosidase in mouse embryos and hematopoietic cells. Proc. Natl. Acad. Sci. USA.

[17] Papapetrou E.P., Schambach A. (2016). Gene Insertion Into Genomic Safe Harbors for Human Gene Therapy. Mol. Ther..

[18] Rodriguez-Fornes F., Quintana-Bustamante O., Lozano M.L., Segovia J.C., Bueren J.A., Guenechea G. (2020). Targeted gene therapy into a safe harbor site in human hematopoietic progenitor cells. Gene Ther..

[19] Pellenz S., Phelps M., Tang W., Hovde B.T., Sinit R.B., Fu W., Li H., Chen E., Monnat R.J. (2019). New Human Chromosomal Sites with “Safe Harbor” Potential for Targeted Transgene Insertion. Hum. Gene Ther..

[20] Vlassis A., Jensen T.L., Mohr M., Jedrzejczyk D.J., Meng X., Kovacs G., Morera-Gómez M., Barghetti A., Muyo Abad S., Baumgartner R.F. (2023). CRISPR-Cas12a-integrated transgenes in genomic safe harbors retain high expression in human hematopoietic iPSC-derived lineages and primary cells. iScience.

[21] Aznauryan E., Yermanos A., Kinzina E., Devaux A., Kapetanovic E., Milanova D., Church G.M., Reddy S.T. (2022). Discovery and validation of human genomic safe harbor sites for gene and cell therapies. Cell Rep. Methods.

[22] Odak A., Yuan H., Feucht J., Cantu V.A., Mansilla-Soto J., Kogel F., Eyquem J., Everett J., Bushman F.D., Leslie C.S., Sadelain M. (2023). Novel extragenic genomic safe harbors for precise therapeutic T-cell engineering. Blood.

[23] Shrestha D., Bag A., Wu R., Zhang Y., Tang X., Qi Q., Xing J., Cheng Y. (2022). Genomics and epigenetics guided identification of tissue-specific genomic safe harbors. Genome Biol..

[24] Xiaozhu Zhang B.V.T., Horton C.A., McIntyre J.J.R., Palm S.M., Shumate J.L., Collins K. (2024). Harnessing eukaryotic retroelement proteins for transgene insertion into human safe-harbor loci. Nat. Biotechnol..

[25] Autio M.I., Motakis E., Perrin A., Bin Amin T., Tiang Z., Do D.V., Wang J., Tan J., Ding S.S.L., Tan W.X. (2024). Computationally defined and in vitro validated putative genomic safe harbour loci for transgene expression in human cells. eLife.

[26] Kimura Y., Shofuda T., Higuchi Y., Nagamori I., Oda M., Nakamori M., Onodera M., Kanematsu D., Yamamoto A., Katsuma A. (2019). Human Genomic Safe Harbors and the Suicide Gene-Based Safeguard System for iPSC-Based Cell Therapy. Stem Cells Transl. Med..

[27] Zhang J., Hu Y., Yang J., Li W., Zhang M., Wang Q., Zhang L., Wei G., Tian Y., Zhao K. (2022). Non-viral, specifically targeted CAR-T cells achieve high safety and efficacy in B-NHL. Nature.

[28] Kikuchi Y., Tamakoshi T., Ishida R., Kobayashi R., Mori S., Ishida-Yamamoto A., Fujimoto M., Kaneda Y., Tamai K. (2023). Gene-Modified Blister Fluid-Derived Mesenchymal Stromal Cells for Treating Recessive Dystrophic Epidermolysis Bullosa. J. Invest. Dermatol..

[29] Zou J., Sweeney C.L., Chou B.K., Choi U., Pan J., Wang H., Dowey S.N., Cheng L., Malech H.L. (2011). Oxidase-deficient neutrophils from X-linked chronic granulomatous disease iPS cells: functional correction by zinc finger nuclease-mediated safe harbor targeting. Blood.

[30] Ordovas L., Boon R., Pistoni M., Chen Y., Wolfs E., Guo W., Sambathkumar R., Bobis-Wozowicz S., Helsen N., Vanhove J. (2015). Efficient Recombinase-Mediated Cassette Exchange in hPSCs to Study the Hepatocyte Lineage Reveals AAVS1 Locus-Mediated Transgene Inhibition. Stem Cell Rep..

[31] Klatt D., Cheng E., Hoffmann D., Santilli G., Thrasher A.J., Brendel C., Schambach A. (2020). Differential Transgene Silencing of Myeloid-Specific Promoters in the AAVS1 Safe Harbor Locus of Induced Pluripotent Stem Cell-Derived Myeloid Cells. Hum. Gene Ther..

[32] Urcelay E., Ward P., Wiener S.M., Safer B., Kotin R.M. (1995). Asymmetric replication in vitro from a human sequence element is dependent on adeno-associated virus Rep protein. J. Virol..

[33] Bertero A., Pawlowski M., Ortmann D., Snijders K., Yiangou L., Cardoso de Brito M., Brown S., Bernard W.G., Cooper J.D., Giacomelli E. (2016). Optimized inducible shRNA and CRISPR/Cas9 platforms for in vitro studies of human development using hPSCs. Development.

[34] Hockemeyer D., Soldner F., Beard C., Gao Q., Mitalipova M., DeKelver R.C., Katibah G.E., Amora R., Boydston E.A., Zeitler B. (2009). Efficient targeting of expressed and silent genes in human ESCs and iPSCs using zinc-finger nucleases. Nat. Biotechnol..

[35] DeKelver R.C., Choi V.M., Moehle E.A., Paschon D.E., Hockemeyer D., Meijsing S.H., Sancak Y., Cui X., Steine E.J., Miller J.C. (2010). Functional genomics, proteomics, and regulatory DNA analysis in isogenic settings using zinc finger nuclease-driven transgenesis into a safe harbor locus in the human genome. Genome Res..

[36] Smith J.R.,S.M., Davis L.A., Alexander M., Yang F., Chandran S., ffrench-Constant C., Pedersen R.A. (2007). Robust, Persistent Transgene Expression in Human Embryonic Stem Cells Is Achieved with AAVS1-Targeted Integration. Stem Cells.

[37] Soucy S.M., Huang J., Gogarten J.P. (2015). Horizontal gene transfer: building the web of life. Nat. Rev. Genet..

[38] Hildebrandt E., Penzes J.J., Gifford R.J., Agbandje-Mckenna M., Kotin R.M. (2020). Evolution of dependoparvoviruses across geological timescales-implications for design of AAV-based gene therapy vectors. Virus Evol..

[39] Penzes J.J., Marsile-Medun S., Agbandje-McKenna M., Gifford R.J. (2018). Endogenous amdoparvovirus-related elements reveal insights into the biology and evolution of vertebrate parvoviruses. Virus Evol..

[40] Kapoor A., Simmonds P., Lipkin W.I. (2010). Discovery and characterization of mammalian endogenous parvoviruses. J. Virol..

[41] Callaway H.M., Subramanian S., Urbina C.A., Barnard K.N., Dick R.A., Bator C.M., Hafenstein S.L., Gifford R.J., Parrish C.R. (2019). Examination and Reconstruction of Three Ancient Endogenous Parvovirus Capsid Protein Gene Remnants Found in Rodent Genomes. J. Virol..

[42] Smith R.H., Hallwirth C.V., Westerman M., Hetherington N.A., Tseng Y.S., Cecchini S., Virag T., Ziegler M.L., Rogozin I.B., Koonin E.V. (2016). Germline viral “fossils” guide in silico reconstruction of a mid-Cenozoic era marsupial adeno-associated virus. Sci. Rep..

[43] Feschotte C., Gilbert C. (2012). Endogenous viruses: insights into viral evolution and impact on host biology. Nat. Rev. Genet..

[44] Wang Y.N., Ye Y., Zhou D., Guo Z.W., Xiong Z., Gong X.X., Jiang S.W., Chen H. (2022). The Role of Syncytin in Placental Angiogenesis and Fetal Growth. Front. Cell Dev. Biol..

[45] Campbell M.A., Loncar S., Kotin R.M., Gifford R.J. (2022). Comparative analysis reveals the long-term coevolutionary history of parvoviruses and vertebrates. PLoS Biol..

[46] Corces M.R., Buenrostro J.D., Wu B., Greenside P.G., Chan S.M., Koenig J.L., Snyder M.P., Pritchard J.K., Kundaje A., Greenleaf W.J. (2016). Lineage-specific and single-cell chromatin accessibility charts human hematopoiesis and leukemia evolution. Nat. Genet..

[47] Mali P., Yang L., Esvelt K.M., Aach J., Guell M., DiCarlo J.E., Norville J.E., Church G.M. (2013). RNA-guided human genome engineering via Cas9. Science.

[48] Oh S.A., Senger K., Madireddi S., Akhmetzyanova I., Ishizuka I.E., Tarighat S., Lo J.H., Shaw D., Haley B., Rutz S. (2022). High-efficiency nonviral CRISPR/Cas9-mediated gene editing of human T cells using plasmid donor DNA. J. Exp. Med..

[49] Bloomer H., Smith R.H., Hakami W., Larochelle A. (2021). Genome editing in human hematopoietic stem and progenitor cells via CRISPR-Cas9-mediated homology-independent targeted integration. Mol. Ther..

[50] Cosenza L.C., Gasparello J., Romanini N., Zurlo M., Zuccato C., Gambari R., Finotti A. (2021). Efficient CRISPR-Cas9-based genome editing of beta-globin gene on erythroid cells from homozygous beta(0)39-thalassemia patients. Mol. Ther. Methods Clin. Dev..

[51] Uchida N., Li L., Nassehi T., Drysdale C.M., Yapundich M., Gamer J., Haro-Mora J.J., Demirci S., Leonard A., Bonifacino A.C. (2021). Preclinical evaluation for engraftment of CD34(+) cells gene-edited at the sickle cell disease locus in xenograft mouse and non-human primate models. Cell Rep. Med..

[52] Rai R., Naseem A., Vetharoy W., Steinberg Z., Thrasher A.J., Santilli G., Cavazza A. (2023). An improved medium formulation for efficient ex vivo gene editing, expansion and engraftment of hematopoietic stem and progenitor cells. Mol. Ther. Methods Clin. Dev..

[53] Six E., Guilloux A., Denis A., Lecoules A., Magnani A., Vilette R., Male F., Cagnard N., Delville M., Magrin E. (2020). Clonal tracking in gene therapy patients reveals a diversity of human hematopoietic differentiation programs. Blood.

[54] Catlin S.N., Busque L., Gale R.E., Guttorp P., Abkowitz J.L. (2011). The replication rate of human hematopoietic stem cells in vivo. Blood.

[55] Radtke S., Pande D., Cui M., Perez A.M., Chan Y.Y., Enstrom M., Schmuck S., Berger A., Eunson T., Adair J.E., Kiem H.P. (2020). Purification of Human CD34(+)CD90(+) HSCs Reduces Target Cell Population and Improves Lentiviral Transduction for Gene Therapy. Mol. Ther. Methods Clin. Dev..

[56] Seczynska M., Bloor S., Cuesta S.M., Lehner P.J. (2022). Genome surveillance by HUSH-mediated silencing of intronless mobile elements. Nature.

[57] Lee B.C., Zhou Y., Bresciani E., Ozkaya N., Dulau-Florea A., Carrington B., Shin T.H., Baena V., Syed Z.A., Hong S.G. (2023). A RUNX1-FPDMM rhesus macaque model reproduces the human phenotype and predicts challenges to curative gene therapies. Blood.

[58] Penaud-Budloo M., Le Guiner C., Nowrouzi A., Chérel Y., Chérel Y., Chenuaud P., Schmidt M., von Kalle C., Rolling F., Moullier P., Snyder R.O. (2008). Adeno-associated virus vector genomes persist as episomal chromatin in primate muscle. J. Virol..

[59] Nopp A., Stridh H., Grönneberg R., Lundahl J. (2002). Lower apoptosis rate and higher CD69 expression in neutrophils from atopic individuals. Inflamm. Res..

[60] Nopp A., Lundahl J., HalldeÂn G. (2000). Quantitative, rather than qualitative, differences in CD69 upregulation in human blood eosinophils upon activation with selected stimuli. Allergy.

[61] Suzukawa M., Komiya A., Yoshimura-Uchiyama C., Kawakami A., Koketsu R., Nagase H., Iikura M., Yamada H., Ra C., Ohta K. (2007). IgE- and FcepsilonRI-mediated enhancement of surface CD69 expression in basophils: role of low-level stimulation. Int. Arch. Allergy Immunol..

[62] Li C., Xiao M., Geng S., Wang Y., Zeng L., Lai P., Gong Y., Chen X. (2024). Comprehensive analysis of human monocyte subsets using full-spectrum flow cytometry and hierarchical marker clustering. Front. Immunol..

[63] Atzeni F., Schena M., Ongari A.M., Carrabba M., Bonara P., Minonzio F., Capsoni F. (2002). Induction of CD69 activation molecule on human neutrophils by GM-CSF, IFN-gamma, and IFN-alpha. Cell. Immunol..

[64] Cunin P., Nigrovic P.A. (2019). Megakaryocytes as immune cells. J. Leukoc. Biol..

[65] Nishikii H., Kurita N., Chiba S. (2017). The Road Map for Megakaryopoietic Lineage from Hematopoietic Stem/Progenitor Cells. Stem Cells Transl. Med..

[66] Calabria A., Spinozzi G., Cesana D., Buscaroli E., Benedicenti F., Pais G., Gazzo F., Scala S., Lidonnici M.R., Scaramuzza S. (2024). Long-term lineage commitment in haematopoietic stem cell gene therapy. Nature.

[67] Cabriolu A., Odak A., Zamparo L., Yuan H., Leslie C.S., Sadelain M. (2022). Globin vector regulatory elements are active in early hematopoietic progenitor cells. Mol. Ther..

[68] Liu M., Maurano M.T., Wang H., Qi H., Song C.Z., Navas P.A., Emery D.W., Stamatoyannopoulos J.A., Stamatoyannopoulos G. (2015). Genomic discovery of potent chromatin insulators for human gene therapy. Nat. Biotechnol..

[69] Ogata T., Kozuka T., Kanda T. (2003). Identification of an insulator in AAVS1, a preferred region for integration of adeno-associated virus DNA. J. Virol..

[70] Shin S., Kim S.H., Shin S.W., Grav L.M., Pedersen L.E., Lee J.S., Lee G.M. (2020). Comprehensive Analysis of Genomic Safe Harbors as Target Sites for Stable Expression of the Heterologous Gene in HEK293 Cells. ACS Synth. Biol..

[71] Yang T.T., Cheng L., Kain S.R. (1996). Optimized codon usage and chromophore mutations provide enhanced sensitivity with the green fluorescent protein. Nucleic Acids Res..

[72] Patro R., Duggal G., Love M.I., Irizarry R.A., Kingsford C. (2017). Salmon provides fast and bias-aware quantification of transcript expression. Nat. Methods.

