# Supplementary material for: Development of evolutionarily conserved viral integration sites as safe harbors for human gene therapy
Source: iScience. 2025 Nov 1;28(12):113910. doi: 10.1016/j.isci.2025.113910 (PMC12719739; doi:10.1016/j.isci.2025.113910)
Supplement: Document S1. Figures S1–S6 and Tables S2–S6 [file mmc1.pdf]

iScience, Volume *28*

Supplemental information

Development of evolutionarily conserved viral integration sites as safe harbors for human gene therapy

Marco A. Quezada-Ramírez, Matthew A. Campbell, Krishna M. Parsi, Robert J. Gifford, and Robert M. Kotin

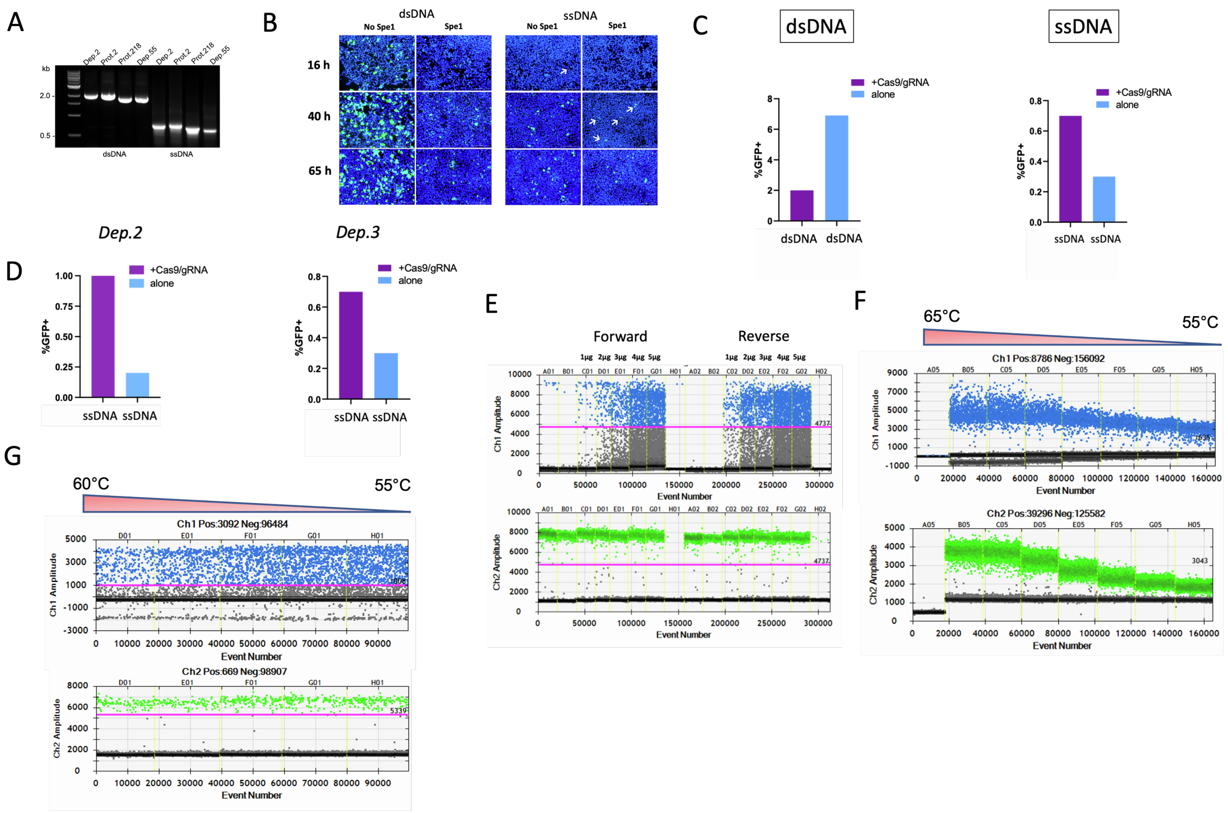

Figure S1. Comparison of dsDNA and ssDNA donor templates for genome editing of human CD34⁺ HSPCs.

A) Representative ssDNA templates (*Dep.2*, *Prot.2*, *Prot.218*, and *Dep.55*) synthesized from the respective DNA duplex substrates. The primers are available in the Key Resources Table. **B)** HEK293 cells transfected with dsDNA, or ssDNA templates, pre-treated with Spe1 restriction enzyme (to eliminate traces of duplex DNA by splitting the transgene into promoter and *eGFP* fragments) reveals that ssDNA takes 40 h to rise detectable GFP signal (arrows). **C)** Two days pre-stimulated CD34⁺ HSPCs were nucleofected with dsDNA (HITI design) or ssDNA donor templates (for HR), both carrying the same MND-eGFP cassette, in presence or absence of CRISPR/Cas9 complexes targeting the *Dep.2* locus. Two days after the nucleofection the GFP⁺ cells were sorted by FACS. Nucleofection with dsDNA alone (i.e., in absence of CRISPR/Cas9 complexes) resulted in a greater number of GFP⁺ cells in absence of CRISPR/Cas9 complexes. In contrast, the use of ssDNA alone showed less percentage of GFP⁺ cells in absence of CRISPR/Cas9 complexes, indicating that ssDNA preparations might prevents the GFP expression from non-integrated templates reducing false positives and the likelihood of random integration, perhaps due to reduced stability of the ssDNA template. **D)** The same phenomenon described in C was observed independently of the targeted locus (*Dep.2* and *Dep.3* are shown). **E)** Nucleofection using dsDNA templates interferes the quantification of edited cells through ddPCR. The samples, processed for ddPCR analysis, displayed “rain” pattern in analyzed samples independently of the amount of dsDNA used for nucleofection (i.e., 1-5 µg). Notice that the primer sets to detect the insertion in forward or reverse orientation (according to the HITI protocol) produce similar

rain pattern. Conversely, primers recognizing an unedited region of the locus (green droplets) produce defined droplet clusters associated to homogeneous amplification. **F)** ddPCR from samples of HSPCs nucleofected with ssDNA templates, targeting the *Dep.2* locus, for junction region (as described in main text) or the reference region generate defined clusters even at different temperatures. **G)** rAAV6-mediated delivery of templates produces rain effect similar to dsDNA-nucleofection (the *Dep.13* locus is shown as example). Notice that the reference amplimer (green) did not show changes under the same temperature gradient.

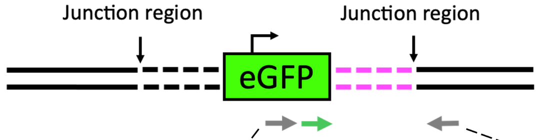


```
Intronic     poly(A)..TCTCCCAGAACCTGAGCTGCTCTGACGCGGCTGTCTGGTGCGTTTCACTGATCCTGGTGCTGCAGCTTCC..  Chr19  AAVS1
Intergenic   poly(A)..AAGGGACACTGCTGTGGTGGTGTTCCCAGACCCAGCAAGACTGACAAGAGTAGGTGCACTAATTGGAAAC..  Chr7   Dep.3
Intronic     poly(A)..NCGAAGAGTTTAGTTAAAATCTTAGTGCACCAGAAGAGTTGCCAAACACCTTTGGTATGGAATTCAAATT..  Chr7   Dep.35
Intergenic   poly(A)..CAAAGNNNTGCTATTAATTCATTAGTGGACTGCAGTTACACATGGN..                         Chr5   Dep.33
Intronic     poly(A)..AAAGTTTATTTAGCTCATAGTTCTAGAGGCTGGAAGTCCACAGTGCCGGCATCTGTGAGGGCCCTTCNNN..  Chr10  Prot.181
Intronic     poly(A)..GGTTTGGACCTTGACTTCCCTCAGTGGATTCGGGGAAATGATTTGACTTACACGCCACTTCTTTGACAAG..  Chr8   Prot.218
Intronic     poly(A)..AAGGAGGAGCCTCCTTCTTGTATTTTAAGGTGTGGGATTATATATGATAAATATTTTATTGTATAGTTTA..  Chr12  Prot.2
Intergenic   poly(A)..AGTATTCTATGTGATTTTAGGCAACTTGGAAGCAAGTAACAGTTAAAAAAGAAGCCCTAGCAAGGGAGCC..  Chr8   Ap.102
Intronic     poli(A)..CCCAGCTCCAGGAAAGACTTGGCACATACCGAGGCTGAAGGTGCTGAATGAAGCAGGCCCCACTTCACAG..  Chr20  Dep.55
Intergenic   poly(A)..TCTCTTCTGCAAATTGGAGACAATGATGCTTCCTGTCTACTAGGGTTGTTATTCTCGGTNTNNAANNNCT..  Chr21  Dep.13
Intronic     poly(A)..CCTTTCTATTANNTAACATGGGGCTGANTGCATTGGCTTACACCTGTAATCCCNNTACTTNNNNAGGCCC..  Chr6   Dep.2
Intronic     poly(A)..AAAATCAAGAATTTGCAGAANNTTTCATATGAGGACATGAGCTTCATTAATCTCTAAAAAATAAGTTTGA..  Chr7   Prot.176
Intronic     poly(A)..GGCACATTTTGAACAACACTGGTCCAAGTTTAATCCATTCTGCTGCTGTGGTCCTATTCCAAAATTCTGT..  Chr9   Dep.1
```

Figure S2. GSH-specific integration of delivered single-stranded DNA templates. Amplicons obtained from edited primary HSPCs (primers are available in Table S3) were gel purified and Sanger-sequenced with a common sequencing primer annealing the rabbit poly(A) signal. All the amplimers started with the poly(A) sequence included in the transgene (green) followed by the right homology arm used for targeted integration (magenta). The arrow indicates the junction between the right homology arm and the targeted locus (black) showing continuity among the sequences.

A

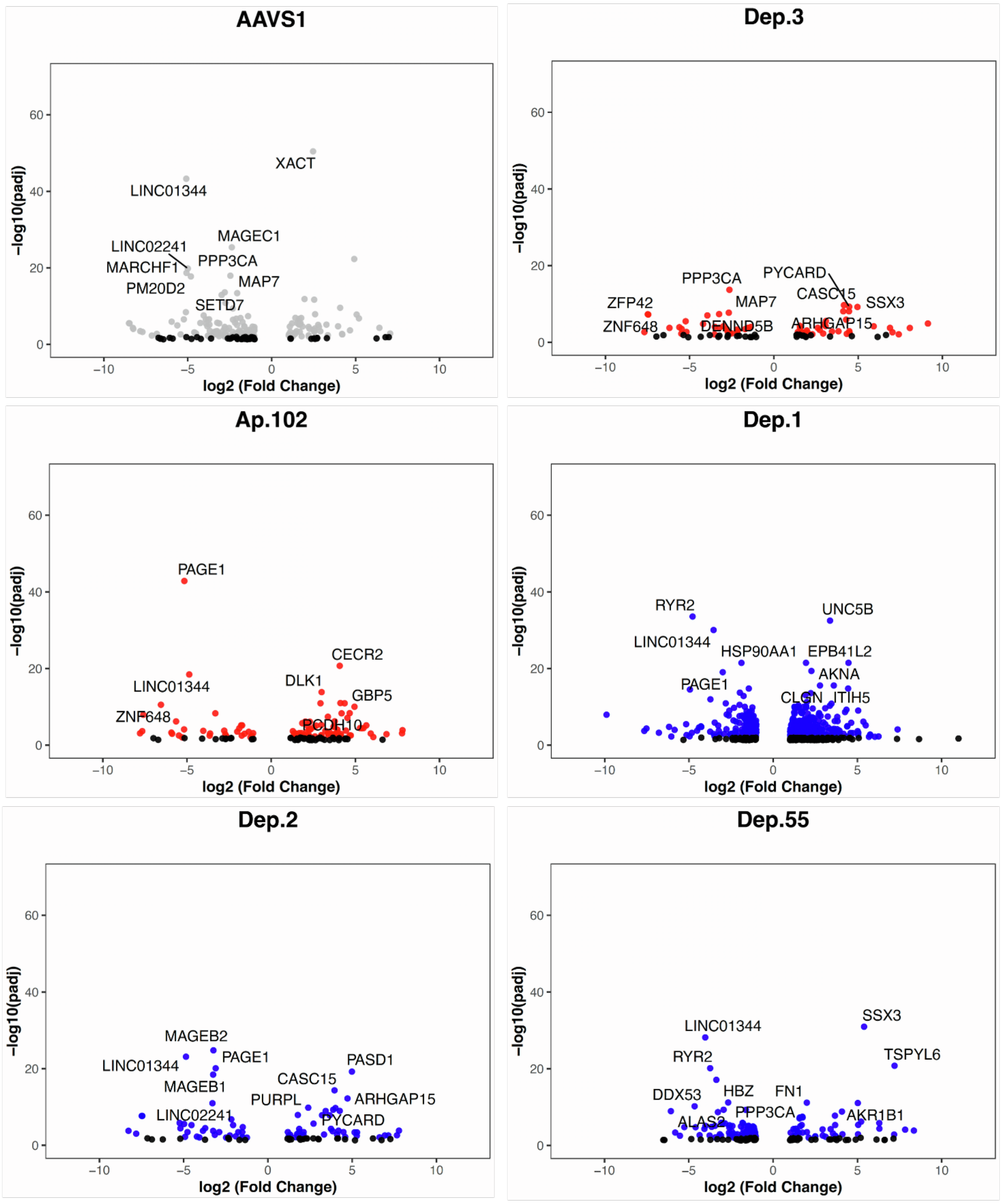
AAVS1
XACT
LINC01344
MAGEC1
LINC02241
PPP3CA
MARCHF1
MAP7
PM20D2
SETD7
Dep.3
PYCARD
PPP3CA
CASC15
ZFP42
MAP7
SSX3
ZNF648
DENND5B
ARHGAP15
Ap.102
PAGE1
CECR2
LINC01344
DLK1
GBP5
ZNF648
PCDH10
Dep.1
RYR2
UNC5B
LINC01344
HSP90AA1
EPB41L2
AKNA
PAGE1
CLGN
ITIH5
Dep.2
MAGEB2
PAGE1
PASD1
LINC01344
CASC15
MAGEB1
PURPL
ARHGAP15
LINC02241
PYCARD
Dep.55
SSX3
LINC01344
TSPYL6
RYR2
DDX53
HBZ
FN1
PPP3CA
ALAS2
AKR1B1
−log10(padj)
log2 (Fold Change)
0
20
40
60
−10
−5
0
5
10

B

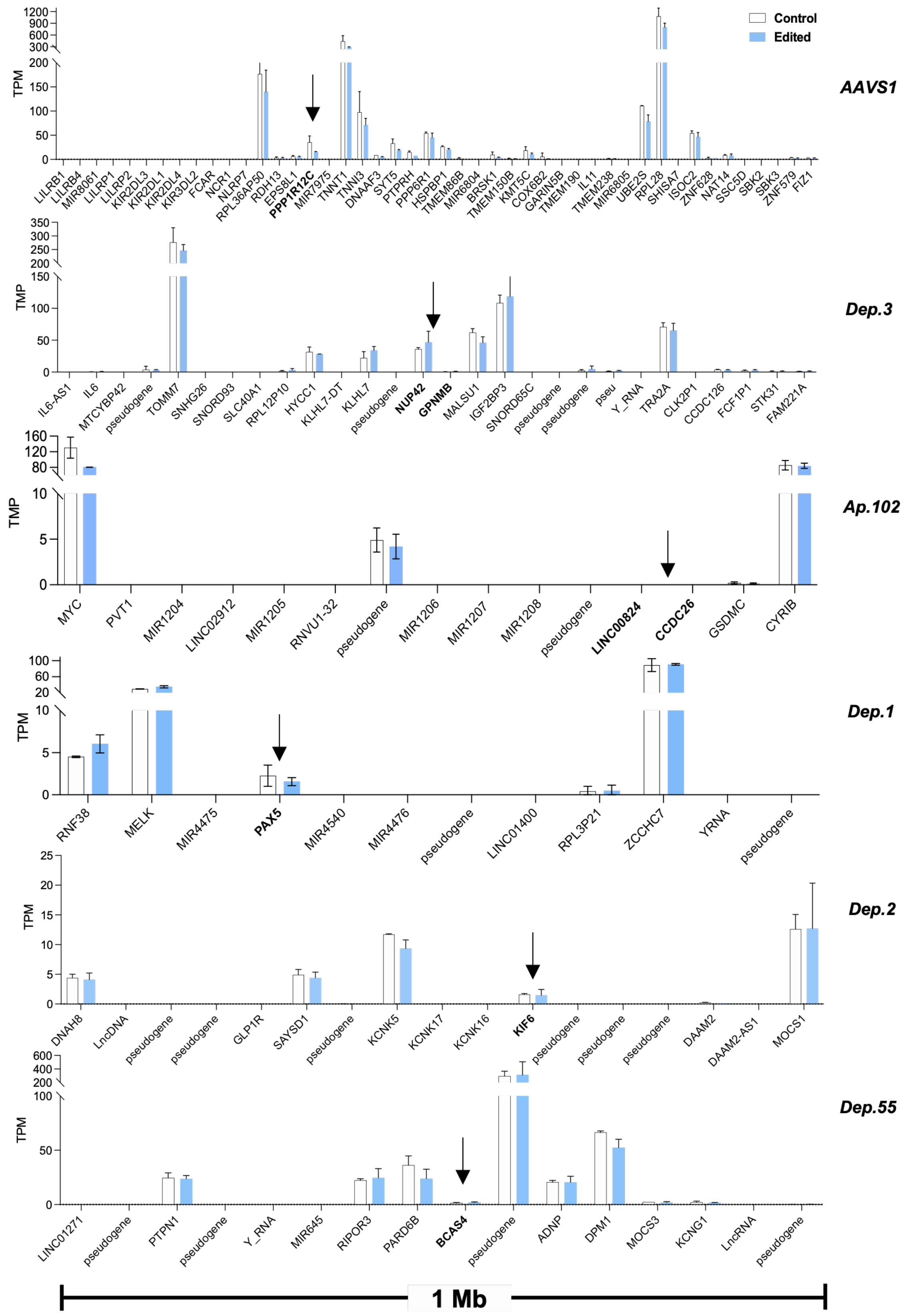

Figure S3. **Gene expression changes determined on knocked-in K562 clones.**
A) Global transcriptional changes after editing of *AAVS1* (63 up-/141 downregulated genes), *Ap.102* (98 up-/42 downregulated genes), *Dep.3* (48 up-/46 downregulated genes), *Dep.1* (472 up-/42 downregulated genes), *Dep.2* (58 up-/42 downregulated genes) or the *Dep.55* locus (75 up-/104 downregulated genes). Genes with adjusted p-values < 0.01 are highlighted in color: gray for *AAVS1*, red for intergenic, and intronic in blue. Highly significant DEGs are indicated. **B)** Transcriptional changes across 1 Mb around the insertion sites (arrows). Transcripts-per-million values represent changes in the expression level before (white bars) and after (blue bars) the gene addition. For intronic GSH sites the host gene is bold. For intergenic GSH sites the flanking genes are bold. Mean ± s.d. *n* = 2 independent K562 clones.

A

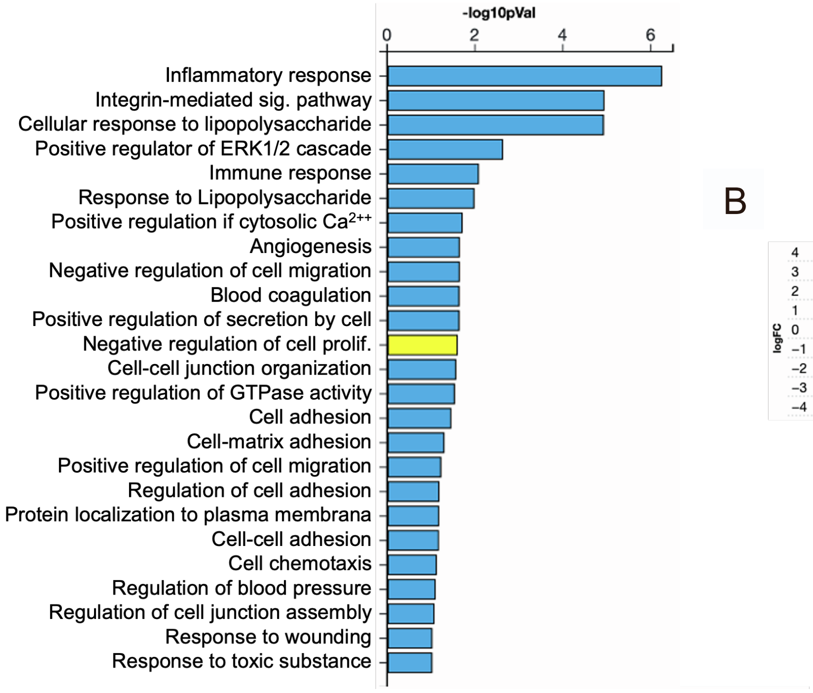

B

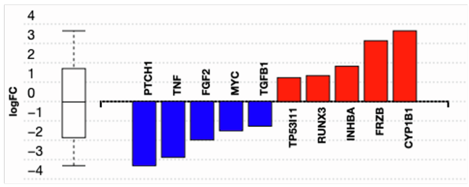

Figure S4. **Gene ontology annotations for *AAVS1*-targeted CD34⁺ cells.**
A) Annotated genes are shown with those associated to cell proliferation highlighted in yellow. **B)** Key tumor associated genes are shown on the right.

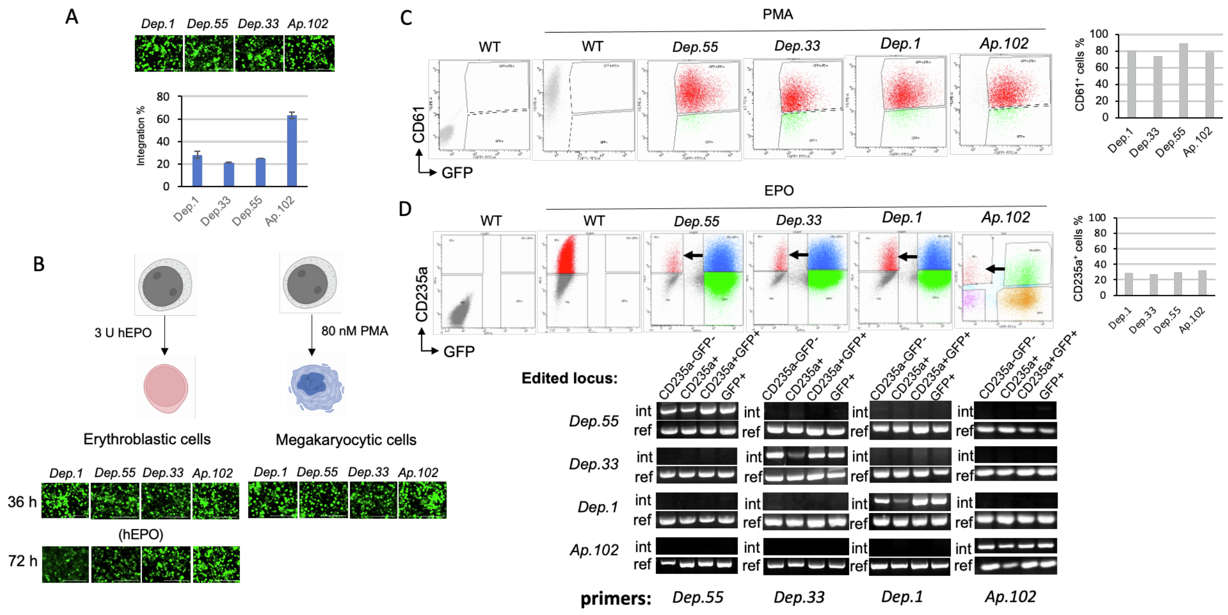

Figure S5. **Erythroid and megakaryocytic induction of *Dep.1*, *Dep.55*, *Ap.102*, or *Dep.33* knocked-in K562 cells.**

A) Pool of stable cells were generated through delivery of ssDNA templates and the appropriated CRIPSR:gRNA complexes. The percentage of edited alleles was determined through ddPCR. **B)** Modified K562 cells were induced to erythroid, or megakaryocytic phenotype, by stimulus with 3U/mL hEPO, or 80 nM PMA, respectively. Representative pictures of induced cells at indicated times are showed. **C)** Cell sorting detected up to 90% of CD61$^+$ megakaryocytic cells with sustained expression of *eGFP* (representative scatterplots and corresponding bar plot are showed to compare to non-induced cells). **D)** Erythroid induction reached just a 30% of CD235a$^+$ cells after 3 days of hEPO induction compared to the non-induced cells. In addition, they showed a less homogeneous phenotype (notice populations in scatterplots). Therefore, the K562 cells induced with hEPO were sorted into the distinct phenotypes detected (i.e., CD235a$^-$GFP$^-$, CD235a$^+$GFP$^-$, CD235a$^+$GFP$^+$, and CD235a$^-$GFP$^+$) and these were analyzed by end point PCR to confirm the presence of the transgene and discard effects of potentially random integrated clones on the observed phenotypes. The presence of the transgene was confirmed in every sorted cell population according to the targeted locus (i.e. *Dep.1*, *Dep.33*, *Dep.55* or *Ap.102*), whereas the reference amplicon (unedited DNA region) was detected independently of the targeted group ("int" refers to integration amplicon, and "ref" corresponds to reference amplicon). These unexpected data confirmed that the transgene was inserted properly in every cell fraction, so It is hard to conclude if in erythroid cells the *eGFP* transgene expression is regulatable (i.e., repressed) by the epigenetic modifications assessed in this report. Nonetheless, this model does not preclude yet the existence of immune specific GSHs given that the expected phenotype CD235a$^+$GFP$^-$ (i.e., erythroid cells where the transgene is silenced) was detected in every experimental group induced with hEPO (arrows in scatterplots). Primers are available in Tables S3 and S4.

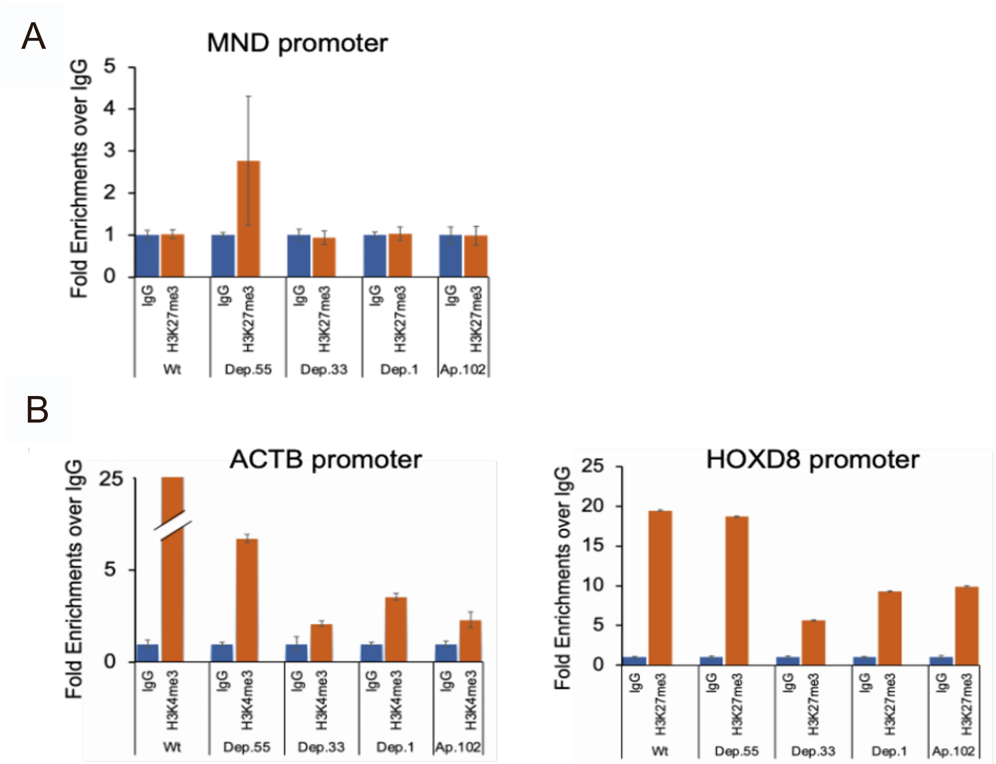

Figure S6. H3K27me3 repressing signal on the MND promoter, and endogenous controls per knocked-in clone to validate the antibodies used.

A) H3K27me3 (repressor) enrichment on MND promoter measured for megakaryocyte induced cells. Interestingly, the epigenetic mark is present when the transgene is inserted into the *Dep.55* locus. **B)** Controls of the epigenetic marks show the enrichment over IgG for H3K4me3 in the *ACTB* locus, and H3K27me3 in the *HoxD8* locus, respectively. Primers are available in Table S6.

Table S2. Candidate GSH loci sustaining the transgene expression through the cell differentiation regardless their relative distance to reported ATAC peaks.

**GSH**	**Category**	**GFP⁺ %**	**P value**	**ATAC peak to**
Dep.3	Intergenic	2.07 ± 0.54	0.8495	0.55 kb
Dep.22	Intergenic	1.32 ± 1.14	0.0258	16.0 kb
Ap.102	Intergenic	0.62 ± 0.41	< 0.0001	29.69 kb
Dep.2	Intronic	3.23 ± 0.86	0.8938	15.0 kb
Prot.181	Intronic	1.35% ± 1.05	0.0473	32.67 kb
Dep.1	Intronic	0.54 ± 0.36	< 0.0001	0.87 kb
Prot.2	Intronic	1.0% ± 0.51	0.0027	1.28 kb
Dep.55	Intronic	1.56 ± 0.53	0.1076	4.0 kb
Prot.218	Intronic	1.62 ± 0.53	0.1961	3.74 kb

Footnote: The percentage of GFP+ cells was determined by FACS sorting at 2 days post-nucleofection (mean ± SD). The nearest ATAC peak (open chromatin) per candidate GSH site was determined with the results informed by Corces M et al. *Nat. Genetics* 48 (2016) for CD34+ HSPCs as described in Table 1 of the main text. The GSH sites within a 2kb distance from a peak are highlighted in blue.

Table S3. Primers to determine GSH-specific integration

Primer name	Sequence 5’ > 3’	Amplicon size (bp)
Universal	GCCTGAGGATCCGATCTTTT	common fwd primer
AAVS1	TCACAGGTAAAACTGACGCA	595
Dep.3	TGAATACCTCTGCAAAGGGC	614
Dep.2	AGGTGGTCTAGAACTCCTGG	533
Dep.34	TGCTCAGTACCTAAGCTTTGG	500
Dep.36	CATTGGCCTCAGTCAGTACC	477
Ap.102	AGTCTTTCTCACTGCTGACA	656
Proto.181	CAAGAAGGCCCTCACAGATG	487
Proto.176	CCTCTGTGTCACTTTCACTGT	534
Dep.35	CGGGTAAATGTTAGGTGCCT	638
Dep.33	ACCATGTGTAACTGCAGTCC	463
Dep.1	GGGTCATTTGTACATGCCCA	535
Proto.1	AGATCCCACCACTGCACT	656
Dep.13	TTCATGCTTGTCAGAGACCG	476
Dep.22	ATGTGAACTGCTGGCCTTTT	475
Dep.28	GAGGTCTTTCTATGGTGCCA	551
Dep.56	ACGGGGTTTCACCATATTGG	513
Proto.218	AAAACGAGCCCATGGAGTTT	560
Dep.55	TGGAGCAGCAGAAATCTCAG	543
Sequencing	CTGCCAAAAATTATGGGGACA	NA

Footnote: The Universal primer is complementary to the poly A sequence of the transgene and was included as a forward primer in all the ddPCR reactions. This oligo is paired with locus specific reverse primers which align outside of the recombination boundary of the targeted locus. The PCR product results uniquely when the locus specific integration had occurred. The Sequencing primer anneals also the rabbit poly(A) signal and was used to confirm the targeted integration of the transgene through Sanger-sequencing of gel-purified amplicons.

.

Table S4. Reference primers recognizing an unedited region of the candidate GSHs

Primer pair name	Sequence 5' > 3'	Targeted sequence	Amplicon (bp)
AAVS1 f	CTCTGACCTGCATTCTCTCC	chr19:55116021-55116564	544
AAVS1 r	TGCCCAAATGAAAGGAGTGA		
Dep.3 f	CAAGCATACATATGGCACGA	chr7:23220274-23220830	557
Dep.3 r	GACCCAGGTCTCCTTTTCTA		
Dep.34 f	AAAGCCGAGACAGGTTGAAA	chr3:96718554-96719078	525
Dep.34 r	GTTTGATGCCTTGAAGCCAG		
Dep.36 f	TTAATGCTTCTGGGAAAGAATTT	chr6:130764868-130765268	401
Dep.36 r	GCCTTGCTTCAATTCCATGA		
Ap.102 f	CTGAAGCAGGAACAGGAGAG	chr8:128435204-128435765	562
Ap.102 r	GCGGGTGTGAGATTTACCAT		
Dep.2 f	GACTCAGGTATCCAACAGCC	chr6:39423568-39424112	545
Dep.2 r	TGGGGGATGGAAGAGAAGAA		
Prot.181 f	TACGAGATAAGGGGCTCCTC	chr10:4652816-4653337	522
Prot.181 r	AACAGGGAGGCTTTTACGAC		
Prot.176 f	CCAGGAACTCTGCATGACAT	chr7:125055768-125056271	504
Prot.176 r	CTTGCCTCTCTCAGAAGCAG		
Dep.35 f	TCCCGAGTAGCTGAGACTAC	chr7:118262291-118262865	575
Dep.35 r	GAGGCATGATTAAGGCACCT		
Dep.33 f	ACTTCAAAGCCATGAACCAT	chr5:50957660-50958167	508
Dep.33 r	GCTTTCTTCTTAAGCTCACCA		
Dep.1 f	CCGATATACACGTACACATGC	chr9:36854330- 36854894	565
Dep.1 r	CGGGAGAAACAGAAGTGAGA		
Prot.2 f	TGGTGTTGCATGCCTGTAAT	chr12:41263414- 41263975	562
Prot.2 r	TCATGGAGTCAGTTTTGGGC		
Dep.13 f	GTCTTTTCTCTCTTCCACAAGA	chr21:32097016-32097537	522
Dep.13 r	ACTGTGAGTCCATTAAACCTC		
Dep.22 f	GCTCCTTCTCTACCACTTTATT	chrx:98174513-98175022	510
Dep.22 r	GAAATGTGACTATTTCATCTATACATT		
Dep.28 f	AGATGCGGGTTTTACCATGT	chr12:40658161- 40658733	573
Dep.28 r	TTGAGATTGCTGCAAATGCC		
Dep.56 f	CTGTTCACCAGCACATTTGT	chr3:108913558-108914068	511
Dep.56 r	TTTAAAATGGAGTCTTGCTTTGT		
Prot.218 f	TGGTGAAAGCCCATGTCTAC	chr8:119592290-119592807	518
Prot.218 r	CTCCTCTTGAGTGGGCTAGA		
Dep.55 f	AGCATCCTGAGTGCATTTCT	chr20:50799100- 50799609	510
Dep.55 r	GGTTGGCTGGACTGAGATTT		

Table S5. Fluorescent probes for ddPCR

Probe name	Sequence 5' > 3'	Fluorophore
Universal	TGAAGCCCCTTGAGCATCTGACTTCTGGCT	FAM
AAVS1	CCCTTGCGTCCCGCCTCCCCT	HEX
Dep.3	ACAGCCAGAGGCAAAAGCTTGCACGC	HEX
Dep.34	CAGCCACAACATCCCCATAAGCCAAAGCCT	HEX
Dep.36	TGTGCAGGGACATAGACCCTCCTCCTCCT	HEX
Ap.102	ACAACCCACCACCGCTATGCAGCAAGA	HEX
Dep.2	CGCACTGCCTGTCCGCAGGCT	HEX
Prot.181	TGCGAAGCTGCCTGGGGCGC	HEX
Prot.176	AGTCCCACGGCATGGATCCTGTGGATAACA	HEX
Dep.35	CCGCCTGCCTCAGCCTCCCA	HEX
Dep.1	GCTCAGGCCAGCCACGAGGGC	HEX
Dep.33	ACCTTCCCGCTGCTGCTGCTGAGT	HEX
Prot.2	ACCACTGCACTCCAGCCTGGGCA	HEX
Dep.13	CCCACCACTCCCCGCCCCCT	HEX
Dep.22	TGCAGCATCTGGCATACTTCCTGGCC	HEX
Prot.181	GCCTGGGGCGCTAGCAGGCA	HEX
Dep.28	GCCACCATGCCTGGCCTCAGT	HEX
Dep.56	TGGGGCTGGGCACGGTGGCT	HEX
Prot.218	ACCCGGGAGGTGGAGGTTGCA	HEX
Dep.55	ACCAGCCTGTCTCCCGGGGGC	HEX

Footnote: The Universal probe (FAM) is complementary to the poly A sequence of the transgene. The HEX probes are specific for an unedited region of the targeted locus. The FAM/HEX ratios were used to determine the level of allelic insertions in GFP^+ cells.

Table S6. Primers for CUT&RUN qPCR

Primer pair name	Sequence 5’ > 3’	Amplicon (bp)
MND promoter f	CCATCCACGCTGTTTTGACC	71
MND promoter r	GCCCTTGCTCACCATGACTA	
ACTB f	CGGGGTCTTTGTCTGAGC	128
ACTB r	CAGTTAGCGCCCAAAGGAC	
HOXD8 f	GGCGAGGCCATCAATCCCAC	101
HOXD 8 r	CTGTTGCCATAGAGCTGCAGG	
